# Comparative analysis of anticholinergic burden scales to explain iatrogenic cognitive impairment in schizophrenia: results from the multicenter FACE-SZ cohort

**DOI:** 10.3389/fphar.2024.1403093

**Published:** 2024-06-12

**Authors:** Nathan Vidal, Paul Roux, Mathieu Urbach, Cristobal Belmonte, Laurent Boyer, Delphine Capdevielle, Julie Clauss-Kobayashi, Thierry D’Amato, Romane Dassing, Caroline Dubertret, Julien Dubreucq, Guillaume Fond, Roxana-Mihaela Honciuc, Sylvain Leignier, Pierre-Michel Llorca, Jasmina Mallet, David Misdrahi, Baptiste Pignon, Romain Rey, Franck Schürhoff, Arnaud Tessier, the FACE-SZ (FondaMental Academic Centers of Expertise—Schizophrenia) Group, B. Aouizerate, Christine Passerieux, Eric Brunet-Gouet

**Affiliations:** ^1^ FondaMental Foundation, Créteil, France; ^2^ Centre Hospitalier de Versailles, Service universitaire de psychiatrie d’adultes et d’addictologie, Le Chesnay, Université Paris-Saclay, Université de Versailles Saint-Quentin-En-Yvelines, DisAP-DevPsy-CESP, Institut National de la Santé et de la Recherche Médicale (INSERM), Villejuif, France; ^3^ University Department of Adult Psychiatry, Hospital La Colombière, CHU Montpellier, Montpellier, France; ^4^ EA 3279: Department of Epidemiology and Health Economics, School of Medicine—La Timone Medical Campus, Marseille University Hospital, Aix-Marseille University, Marseille, France; ^5^ Institute of Functional Genomics, University of Montpellier, Centre National de la Recherche Scientifique (CNRS), Institut National de la Santé et de la Recherche Médicale (INSERM), Montpellier, France; ^6^ Department of Psychiatry, University Hospitals of Strasbourg, University of Strasbourg, Institut National de la Santé et de la Recherche Médicale (INSERM), Strasbourg, France; ^7^ Le Vinatier Hospital, Schizophrenia Expert Centre, Institut National de la Santé et de la Recherche Médicale (INSERM), Centre National de la Recherche Scientifique (CNRS), University Lyon 1, Lyon Neuroscience Research Center, PSYR2 Team, Lyon, France; ^8^ Assistance Publique—Hôpitaux de Paris (AP-HP), Department of Psychiatry, Louis Mourier Hospital, Institut National de la Santé et de la Recherche Médicale (INSERM), Institute of Psychiatry and Neuroscience of Paris, University Paris Descartes, Université Paris Diderot, Sorbonne Paris Cité, Faculté de Médecine, Paris, France; ^9^ Grenoble Alpes University, Institut National de la Santé et de la Recherche Médicale (INSERM), CHU Grenoble Alpes, Grenoble Institute of Neurosciences, Grenoble, France; ^10^ CHU Clermont-Ferrand, Service of psychiatry B, University of Clermont Auvergne, Clermont-Ferrand, France; ^11^ Department of Universitary and General Psychiatry, Charles Perrens Hospital, University of Bordeaux, Aquitaine Institute for Cognitive and Integrative Neuroscience (CNRS UMR 5287-INCIA, ECOPSY), Bordeaux, France; ^12^ Assistance Publique—Hôpitaux de Paris (AP-HP), Hôpitaux Universitaires “H. Mondor”, DMU IMPACT, Institut National de la Santé et de la Recherche Médicale (INSERM), Institut Mondor de Recherche Médicale (IMRB), Translational Neuropsychiatry, University Paris-Est-Créteil (UPEC), Créteil, France

**Keywords:** neuropsychological test, schizophrenia, cholinergic antagonist, psychotropic drug, polypharmacy

## Abstract

**Aim:**

The anticholinergic properties of medications are associated with poorer cognitive performance in schizophrenia. Numerous scales have been developed to assess anticholinergic burden and yet, there is no consensus indicating which anticholinergic burden scale is more relevant for patients with schizophrenia. We aimed to identify valid scales for estimating the risk of iatrogenic cognitive impairment in schizophrenia.

**Methods:**

We identified 27 scales in a literature review. The responses to neuropsychological tests of 839 individuals with schizophrenia or schizoaffective disorder in the FACE-SZ database were collected between 2010 and 2021. We estimated the association between objective global cognitive performance and the 27 scales, the number of psychotropic drugs, and chlorpromazine and lorazepam equivalents in bivariable regressions in a cross-sectional design. We then adjusted the bivariable models with covariates: the predictors significantly associated with cognitive performance in multiple linear regressions were considered to have good concurrent validity to assess cognitive performance.

**Results:**

Eight scales, the number of psychotropic drugs, and drug equivalents were significantly associated with cognitive impairment. The number of psychotropic drugs, the most convenient predictor to compute, was associated with worse executive function (Standardized β = −0.12, *p* = .004) and reasoning (Standardized β = −0.08, *p* = .037).

**Conclusion:**

Anticholinergic burden, the number of psychotropic drugs, and drug equivalents were weakly associated with cognition, thus suggesting that cognitive impairment in schizophrenia and schizoaffective disorder is explained by factors other than medication. The number of psychotropic drugs was the most parsimonious method to assess the risk of iatrogenic cognitive impairment.

## 1 Introduction

Schizophrenia spectrum disorders (SZ) are associated with cognitive impairment (Schaefer et al., 2013), marked by significant deficits in attention, learning, memory, executive function, and social cognition (Green et al., 2000). Pharmacological treatments may exacerbate cognitive impairment in SZ; while antipsychotics exhibit heterogeneous effects on cognition (Baldez et al., 2021), tricyclic antidepressants (Podewils and Lyketsos, 2002) and antiparkinsonian agents that alleviate extrapyramidal side effects are known to be associated with poorer cognitive performance (Brébion et al., 2004). In addition, reducing the use of antiparkinsonian agents is associated with better cognitive performance in SZ (Desmarais et al., 2014). These results suggest that the anticholinergic properties of psychotropic drugs, which consist of inhibitory activity on acetylcholine receptors, contribute to cognitive impairment in SZ.

Anticholinergic burden scales aim to assess the anticholinergic properties of medications by attributing an anticholinergic score to each drug for the entire prescription. Anticholinergic burden scales preferably include medications used by the elderly, such as cyclobenzaprine or atorvastatin (Carnahan et al., 2006). It stems from the fact that most scales were developed to assess the anticholinergic burden in the elderly population, which is particularly vulnerable to anticholinergic side effects (Lisibach et al., 2021). Nevertheless, the scales also include medications commonly prescribed in psychiatry, such as clozapine or amitriptyline (Carnahan et al., 2006), thus enabling their application in this field. Several studies have reported a significant association between the scores on anticholinergic burden scales and poorer cognitive performance in SZ (Georgiou et al., 2021), highlighting the validity of these scales in assessing the risk of iatrogenic cognitive impairment. More specifically, among subjects with SZ, anticholinergic burden scores are associated with worse performance in working memory (Minzenberg et al., 2004; Ang et al., 2017; Joshi et al., 2021; Verdoux et al., 2021), verbal memory (Minzenberg et al., 2004; Eum et al., 2017; Ballesteros et al., 2018; Joshi et al., 2019; Joshi et al., 2021; Haddad et al., 2023), and, to a lesser extent, executive function, attention, and processing speed (Ang et al., 2017; Joshi et al., 2021).

Most studies that examined the association between anticholinergic burden scores and cognitive performance controlled for potential confounding variables, such as sex, age, and the severity of symptoms (Ang et al., 2017; Eum et al., 2017; Ballesteros et al., 2018; Joshi et al., 2019; Joshi et al., 2021; Verdoux et al., 2021; Haddad et al., 2023). Negative symptoms of schizophrenia, known to be associated with significant cognitive impairment (Harvey et al., 2006; Ventura et al., 2009), and positive symptoms, which are linked to poorer social cognition (Peyroux et al., 2019), are often considered in this context. Indeed, more intense symptoms may require higher doses of antipsychotics, which could, in turn, lead to a spurious association between medication and cognition (Faber et al., 2012). Two factors associated with impaired cognitive performance that can potentially lead to additional antipsychotic prescriptions and thus higher anticholinergic burden are multiple hospitalizations (Goldberg et al., 2011) and psychotic episodes (Braw et al., 2008; Corigliano et al., 2014). However, the consideration of these two factors has been limited to only a few studies. In addition, individuals with schizoaffective disorder, who are more likely to use antidepressants than individuals with schizophrenia, may experience an increased anticholinergic burden (Olfson et al., 2009). Surprisingly, one study (Minzenberg et al., 2004) focusing on people with schizophrenia did not incorporate potential confounding variables during the design and testing of their scales. It is essential to assess the contribution of variables such as symptom severity, past hospitalizations, psychotic episodes, schizophrenia subtype, and socio-demographic factors to the association between the anticholinergic burden and cognition in schizophrenia. Furthermore, the inclusion of different sets of covariates between studies complicates the identification of the most relevant scale(s). Comparing multiple anticholinergic burden scales while adjusting for the same set of covariates appears to be the optimal method for evaluating their validity. For example, Ang et al. (Ang et al., 2017) compared the validity of two scales to predict iatrogenic cognitive impairment while controlling for sex, age, and the duration and severity of the illness.

However, the authors relied on two scales, whereas at least 22 different scales were available at the beginning of the present study (Lisibach et al., 2021), and they differed substantially (Rudd et al., 2005). Indeed, some scales were designed based on expert-driven literature reviews of the anticholinergic properties of the drugs (Rudolph et al., 2008), whereas others were based exclusively on objective *in vitro* measurements of the serum anticholinergic activity of the drugs (Chew et al., 2008). As a result, a single drug can be classified as highly anticholinergic on one scale and as non-anticholinergic on another. For example, baclofen is moderately anticholinergic on the Anticholinergic Risk Scale (Rudolph et al., 2008) but is not on Chew’s scale (Chew et al., 2008). Although recent scores tend to converge (Al Rihani et al., 2021), the significant differences between scales underscore the need to identify valid scales to assess iatrogenic cognitive impairment in SZ. Indeed, a consensus has yet to be reached concerning the most relevant scale(s) to use in SZ.

Our primary objective was to identify all available scales based on a literature review. We then wanted to determine which scales were associated with the iatrogenic cognitive burden in individuals with SZ to establish their concurrent validity in assessing iatrogenic cognitive deficit. To ensure that the putative associations between cognitive performance and anticholinergic scores were driven by iatrogenic side effects, several of the clinical factors mentioned above were introduced as adjustment variables. In addition, we investigated whether the scales exhibit stronger associations with cognitive performance versus alternative treatment-dependent variables that correlate with cognitive impairment, such as the number of psychotropic drugs (Chakos et al., 2006) or chlorpromazine equivalents (Ballesteros et al., 2018). Ultimately, our goal was to recommend a tool that effectively identifies individuals with SZ at a higher risk of additional cognitive impairment.

## 2 Materials and methods

The study preregistration is available at https://osf.io/r3h4g/?view_only=e744e576d2c942708b9bacec4eeb5768.

### 2.1 Study design and characteristics of the recruiting network

This study was conducted in multiple centers and included patients of the FACE-SZ cohort, which is a part of the FondaMental Advanced Centers of Expertise for Schizophrenia. The cohort was recruited between 2010 and 2021 through a network of 10 centers located in Bordeaux, Clermont-Ferrand, Colombes, Créteil, Grenoble, Lyon, Marseille, Montpellier, Strasbourg, and Versailles established by the Fondation FondaMental (https://www.fondation-fondamental.org) under the French Ministry of Research. The study received approval from the local ethics committee, known as the Comité de Protection des Personnes Ile de France IX, on 18 January 2010, according to the regulations for non-interventional studies in France. Non-interventional studies refer to observational studies that do not involve any additional or unusual procedures related to diagnosis, treatment, or monitoring and pose no risks or constraints. Although written informed consent was not required, all patients received an informational letter, and verbal consent was obtained and documented officially. We used the data from the first visit of the patients to the Centers of Expertise for Schizophrenia.

### 2.2 Participants

The diagnosis of SZ was determined using the criteria outlined by First et al. (First et al., 2016) in the Structured Clinical Interview for DSM-5 (SCID). We included 18- to 65-year-old outpatients diagnosed with schizophrenia or schizoaffective disorder. We excluded patients with a history of neurological disorders, dyslexia, dyscalculia, dysphasia, dysorthographia, or dyspraxia, those presenting any symptoms of substance dependence over the past month, and those who had received electro-convulsive therapy within the past year, thus eliminating the known factors unrelated to medication that could contribute to cognitive impairment.

### 2.3 Measurements

#### 2.3.1 Exposure: anticholinergic burden

We conducted a literature review using Google Scholar, PubMed, and the Cochrane library to identify anticholinergic burden scales published before 24 November 2022 (see Supplementary Material Sl for more details).

For drugs that were not included in a scale, we assigned a score of 0, indicating that they had no anticholinergic properties based on the scale, following a similar approach as in a previous study (Lisibach et al., 2022). To calculate the overall anticholinergic burden of the treatment, we used two different methods. The first involved summing the scores of all relevant drugs (“sum”) according to the scale, as described in a study by Carnahan et al. (Carnahan et al., 2006) The second used the highest score among the drugs (“max”), following the approach outlined in a study by Sittironnarit et al. (Sittironnarit et al., 2011).

#### 2.3.2 Outcome: cognition

The tests were administered by neuropsychologists following a predetermined sequence in each center. The duration of the testing session was approximately 120 min, including short breaks of between 5 and 10 min. The neurocognitive domains investigated by the neuropsychological test battery were:

- Processing speed, evaluated using the digit symbol coding subtest from the Wechsler Adult Intelligence Scale (WAIS) version III (Wechsler, 1997) or the coding subtest from the WAIS-IV (Wechsler et al., 2008), the Trail Making Test (TMT) part A (Reitan, 1958), and verbal fluency (semantic and phonemic) (Lezak, 2004).
- Attention, evaluated using the Continuous Performance Test-identical pairs version (CPT-IP) (Cornblatt et al., 1988) and the alertness, flexibility, divided attention, and go/no-go tests of the Test of Attentional Performance (TAP) (Zimmermann and Fimm, 2002).
- Working memory, evaluated using the digit span, arithmetic, and digit-letter sequencing WAIS subtests (version III or IV).
- Verbal memory, evaluated using the California Verbal Learning Test (CVLT) (Delis, 2000).
- Visual memory, evaluated using the doors test (Baddeley et al., 2006).
- Reasoning, evaluated using matrix reasoning and picture completion (WAIS-III or IV) for perceptual reasoning and similarities (WAIS-III or IV) for verbal reasoning.
- Executive functioning, evaluated using the TMT part B and the Modified Six Elements Test (Wilson et al., 1997).

Raw scores were transformed into demographically corrected z-scores based on normative data for each test (Zimmermann and Fimm, 2002; Poitrenaud et al., 2007; Godefroy, 2008; Kern et al., 2008; Sittironnarit et al., 2011). Higher scores reflect better performance. We computed a mean z-score for each cognitive domain and averaged them to compute a global cognition score.

#### 2.3.3 Clinical covariates and alternative predictors of iatrogenic cognitive burden

Socio-demographic factors (sex, age, education level), the total number of psychotic episodes, the number of hospitalizations, and the subtype of SZ (schizophrenia or schizoaffective disorder) were collected. These were all considered potential covariates in the assessment of the association of anticholinergic burden with cognitive impairment.

The severity of symptoms was evaluated using the Clinical Global Impression-Severity scale (CGI-S) (Guy, 1976), which is a clinician-rated scale. A high score on the CGI-S indicates greater symptom severity. Schizophrenic symptomatology was assessed using the total score of the Positive and Negative Syndrome Scale (PANSS) (Kay et al., 1987). Depressive symptoms were evaluated using the Calgary Depression Rating Scale for Schizophrenia (Addington et al., 1992). Scores from the CGI-S, Calgary Depression Rating Scale, and PANSS positive and negative symptom subscores were also screened as potential covariates.

Additional information included the age at the first episode and at the first treatment. The class of treatment (antidepressants, anticonvulsants, lithium, antipsychotics, anxiolytics, or antiparkinsonian drugs prescribed for extrapyramidal side effects) was recorded. We distinguished between the use of first-generation antipsychotics and atypical or second-generation antipsychotics, as classified by the US Food and Drug Administration (Supplementary Figure S1).

Four alternative correlates of iatrogenic cognitive burden were collected, namely, the number of psychotropic drugs (Chakos et al., 2006) (including antipsychotics, antidepressants, anxiolytics, antiparkinsonian drugs, mood stabilizers, and hypnotics), the number of antipsychotics (Élie et al., 2010), chlorpromazine equivalents (Ballesteros et al., 2018) (CPZeq, computed from the formulas proposed by Andreasen et al. (Andreasen et al., 2010) and Leucht et al. (Leucht et al., 2015)), and lorazepam equivalents (Savić et al., 2021) (based on the formulas proposed by Kane et al. (Kane, 2017)). We estimated the association between these alternative measurements and cognitive performance.

### 2.4 Statistical analysis

Statistical analyses were carried out using R version 4.3.0. First, we conducted successive bivariable linear regressions on the complete cases to examine the relationships between the 27 anticholinergic scale scores and the global cognition score. In addition, we performed successive bivariable linear regressions using the four alternative correlates of iatrogenic cognitive burden (the number of psychotropic drugs, antipsychotics, CPZeq, and lorazepam equivalents).

Subsequently, we carried out multiple linear regressions of the global cognition score for predictors significantly associated with the global cognition score at a 5% level in the bivariable linear regressions by adjusting the models for a subset of covariates. The variables that could confound the association between anticholinergic burden and cognitive performance were screened as potential covariates. They were then selected as covariates if they were associated with the scale validated by the most studies, i.e., the Anticholinergic Cognitive Burden scale (Boustani et al., 2008; Lisibach et al., 2021), with a *p*-value ≤0.2 (see the covariate selection process in Supplementary Material S2 and Supplementary Table S1).

For the multiple analyses, we considered data missing at random (MAR). We estimated missing data using multivariate imputation by chained equations (50 imputations, *mice* package (Van Buuren et al., 2011) of R, version 3.15.0). Each covariate had <30% missing data, which enabled us to use multiple imputations (Marshall et al., 2010). To ensure the reliability of the imputed values, we compared the imputed and non-imputed datasets (Nguyen et al., 2017). We report the fraction of missing information (fmi) computed using the *pool* function of the *mice* package in the results.

In addition, we conducted multiple linear regressions of the z-score in each cognitive domain (processing speed, visual memory, verbal memory, attention, working memory, executive function, and reasoning) and the most pertinent predictors that were significantly associated with global cognitive performance in the multiple regression models. We used the same set of covariates as above and reported the standardized coefficients estimated by the models.

## 3 Results

### 3.1 Description of the sample

We included 839 adults with SZ in the final sample (26% female) (Table 1). The mean age was 31.6 years (SD_age_ = 9.3 years). The actual sample size was higher than estimated in our power analysis (see the preregistration). In addition, we identified 36 anticholinergic burden scales (information about the scales is reported in Supplementary Material S1). We discarded two scales because they excluded psychotropic medications, four because we selected a revised and more recent version instead, one that did not exclusively evaluate anticholinergic properties, and two that were unavailable. We selected the most recent version of each of the remaining 27 scales. The anticholinergic scales reported between 10.5% and 78.9% of patients with a non-zero score, i.e., with an anticholinergic burden, and between 4.5% and 56.6% with a high anticholinergic burden (the thresholds defining a high anticholinergic burden are explained in Supplementary Material S3), suggesting a large discrepancy between scales (Supplementary Figure S2). The cognitive performance of the sample is presented in Table 2.

**TABLE 1:** Description of the sample (n = 839).

Category	n	Mean (SD)	n (%) missing data
Female, n (%)	214 (26%)		0
Age (mean, ±SD)		31.6 (9.3)	0
Education level (years)		12.5 (2.3)	15 (2%)
PANSS total score (mean, ±SD)		69.5 (19.5)	57 (7%)
PANSS positive score (mean, ±SD)		14.6 (5.9)	52 (6%)
PANSS negative score (mean, ±SD)		20.2 (7.3)	52 (6%)
PANSS general psychopathology score (mean, ±SD)		34.8 (10.2)	55 (7%)
Calgary (mean, ±SD)		3.9 (4.1)	47 (6%)
Schizophrenia, n (%)	666 (79%)		0
Schizoaffective disorder, n (%)	173 (21%)		0
CGI-S (mean, ±SD)		4.4 (1.1)	38 (5%)
Number of psychotic episodes (mean, ±SD)		3 (4)	140 (17%)
Age at the first psychotic episode (mean, ±SD)		21.4 (6)	59 (7%)
Number of hospitalization (mean, ±SD)		4 (4)	154 (18%)
Age at first treatment (mean, ±SD)		22.6 (6.4)	76 (9%)
Number of psychotropic drugs (mean, ±SD)		2.4 (1.4)	173 (21%)
Number of antipsychotics (mean, ±SD)		1.3 (0.6)	173 (21%)
Chlorpromazine equivalents, mg/24 h (mean, ±SD)		548 (416)	213 (25%)
Lorazepam equivalents, mg/24 h (mean, ±SD)		0.24 (0.65)	173 (21%)
Patients taking antipsychotics, n (%)	648 (97%)		173 (21%)
…including first-generation antipsychotics, n (%)	166 (25%)		173 (21%)
…including second-generation antipsychotics, n (%)	608 (91%)		173 (21%)
Patients taking antidepressants, n (%)	160 (24%)		173 (21%)
…anxiolytics, n (%)	151 (23%)		173 (21%)
…antiparkinsonian drugs, n (%)	106 (16%)		173 (21%)
…mood stabilizer, n (%)	100 (15%)		173 (21%)
…hypnotics, n (%)	54 (8%)		173 (21%)

PANSS: the Positive and Negative Syndrome Scale.

Calgary: the Calgary Depression Rating scale.

CGI: Clinical Global Impression—Severity scale.

**TABLE 2:** Cognitive performance in the seven cognitive domains and over all domains (n = 839).

Category	Mean	SD	n (%) missing data
Attention (mean, ±SD)	−0.84	0.80	306 (37%)
Executive function (mean, ±SD)	−0.97	1.04	105 (13%)
Processing speed (mean, ±S D)	−0.87	0.81	90 (11%)
Reasoning (mean, ±SD)	−0.48	1.04	117 (14%)
Verbal memory (mean, ±SD)	−0.98	1.04	140 (17%)
Visual memory (mean, ±SD)	−1.10	1.11	374 (45%)
Working memory (mean, ±SD)	−0.62	0.84	112 (13%)
Global cognition score (mean, ±SD)	−0.83	0.70	73 (9%)

Note: Values represent demographically corrected z-scores based on normative data.

### 3.2 Global cognitive performance and anticholinergic burden scales

**FIGURE 1:**
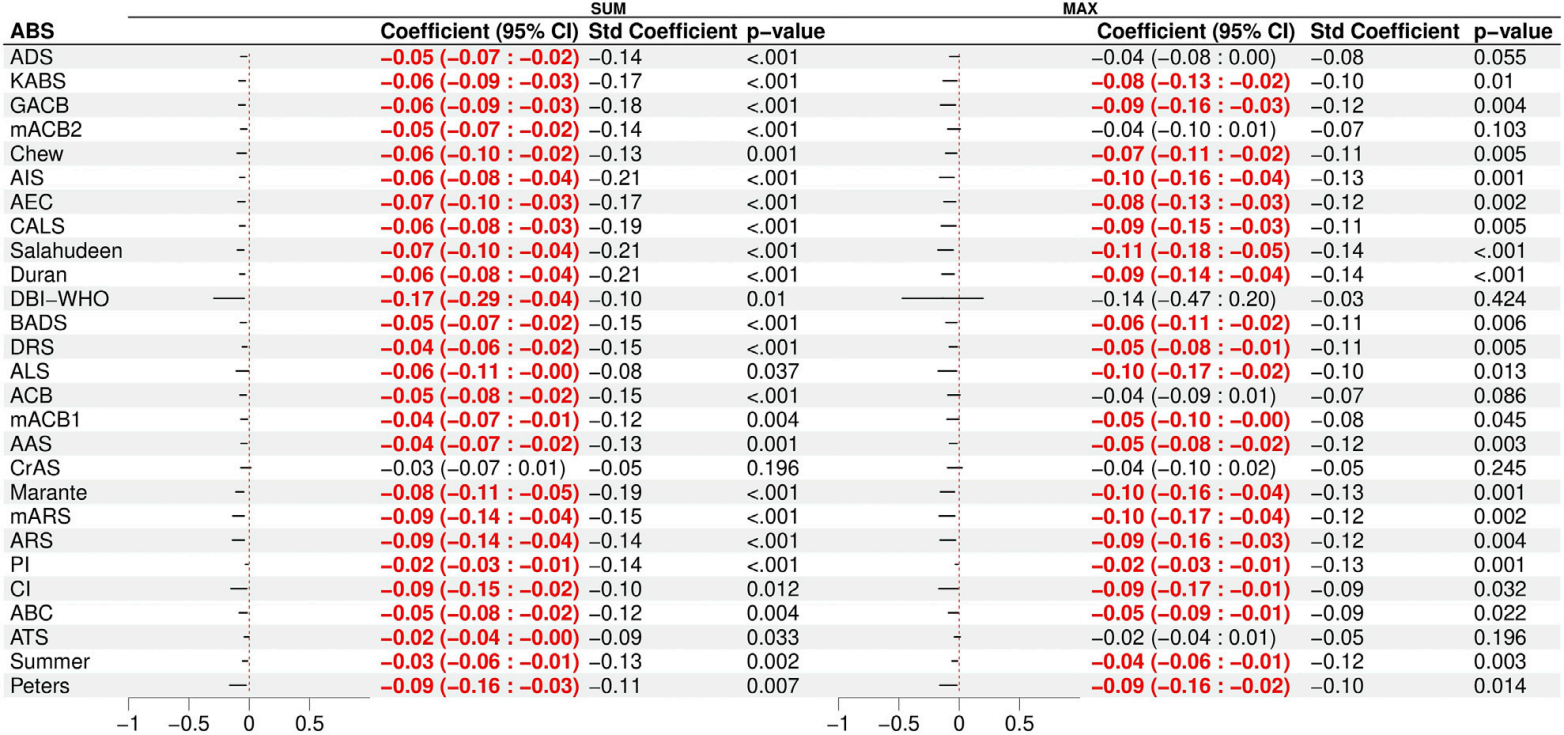
Results of the bivariable linear regression models of cognitive impairment with the 27 scales as the predictor. Significant (*p* < 0.05) associations are shown in red. The total anticholinergic burden score was computed by either summing the scores of each treatment (SUM) or by using the maximum score (MAX).

The results of bivariable regressions, which examined the association between the anticholinergic burden scales and global cognitive performance, are presented in Figure 1. The scores of 26 scales were significantly associated with a decrease in the global cognition score when using the “sum” method (−0.09 ≤ Standardized β ≤ −0.21), while the scores of 21 scales were significantly associated with a decrease in the global cognition score when using the “max” method (−0.08 ≤ Standardized β ≤ −0.14). We adjusted the bivariable models of these scales using a set of covariates (see the covariate selection process in Supplementary Material S2). After adjusting for the covariates, eight of the 26 scales were still significantly associated with cognitive impairment in multiple linear regressions (Supplementary Table S2). The eight scales were the Anticholinergic Cognitive Burden scale (Boustani et al., 2008), the Anticholinergic Effect on Cognition scale (Bishara et al., 2017), the Anticholinergic Impregnation Scale (Briet et al., 2017), the CRIDECO Anticholinergic Load Scale (Ramos et al., 2022), Durán’s scale (Durán et al., 2013), the German Anticholinergic Burden scale (Kiesel et al., 2018), the Korean Anticholinergic Burden Scale (Jun et al., 2019), and Salahudeen’s scale (Salahudeen et al., 2015), all computed using the sum method. The number of psychotropic drugs (standardized β = −0.1, *p* = .016), CPZeq (standardized β = −0.11, *p* = .005), and lorazepam equivalents (standardized β = −0.09, *p* = .037) also showed a significant association with lower cognitive performance after adjusting for the same set of covariates (Supplementary Table S2), unlike the number of antipsychotics, which did not. Given that the number of psychotropic drugs is easier to calculate than drug dose equivalents or anticholinergic burden scales, these findings suggest that the number of psychotropic drugs is the most convenient method to evaluate iatrogenic cognitive burden.

Among the covariates, the PANSS negative score and the severity score of the Clinical Global Impression scale were consistently significantly associated with a decrease in global cognitive performance in all multiple regression models (for the PANSS: standardized β = −0.19; for the CGI-S: 0.17 ≤ standardized β ≤ −0.19).

### 3.3 Performance in cognitive domains and the number of psychotropic drugs

Because the number of psychotropic drugs emerged as the most convenient indicator of iatrogenic cognitive burden, we conducted multiple linear regressions for each cognitive domain using the number of psychotropic drugs as the primary predictor and adjusting for the covariates. The standardized coefficients of these models are reported in Table 3. The number of psychotropic drugs was associated with poorer performance in executive function (standardized β = −0.11, *p* = .007) and reasoning (standardized β = −0.1, *p* = .014).

**TABLE 3:** Standardized coefficients of the associations between the number of medications and each separated cognitive domain in multiple linear regression models. For the sake of clarity, the coefficients of the covariates are not represented. Significant results are indicated in bold.

Cognitive domain	Number of medications	
	Standardized coefficient	*p*-value
Executive function	**−0.11**	**0.007**
Processing speed	−0.03	0.392
Verbal memory	−0.02	0.602
Attention	−0.07	0.127
Working memory	−0.08	0.050
Reasoning	**−0.10**	**0.014**
Visual memory	−0.06	0.289

## 4 Discussion

We assessed the concurrent validity of 27 anticholinergic burden scales to assess cognitive impairment in a large cohort of outpatients with SZ. Between 4.5% and 56.6% of our sample was considered to have a high anticholinergic burden, underscoring the importance of assessing the risk of anticholinergic burden in SZ.

We identified eight scales with good concurrent validity in assessing cognitive impairment in SZ. The scores of those eight scales were associated with cognitive impairment, even after adjusting for symptom severity, the number of past psychotic episodes, the number of past hospitalizations, and the subtype of SZ. The eight scales include scores that were associated with lower cognitive performance in schizophrenia in previous studies, such as the Anticholinergic Cognitive Burden scale (Ang et al., 2017; Joshi et al., 2019) and Salahudeen’s scale (Salahudeen et al., 2015). In addition, the Anticholinergic Impregnation Scale, a French scale designed to evaluate the anticholinergic burden in psychiatry (Briet et al., 2017; Javelot et al., 2022), had never been validated for people with schizophrenia before. By contrast, certain scales, such as the Anticholinergic Drug Scale (Ang et al., 2017; Eum et al., 2017; Haddad et al., 2023), the Anticholinergic Risk Scale (Ballesteros et al., 2018), the Pharmacological Index (Minzenberg et al., 2004), the Anticholinergic Cognitive Burden scale version of Joshi et al. (Joshi et al., 2021) (called mACB2 in our study), the Clinical index (Minzenberg et al., 2004), and the Drug Burden Index (Cuesta et al., 2022), were expected to be significantly associated with cognitive performance in multiple regression models. This discrepancy can be attributed to differences in the sample tested or in the list of covariates. For example, Minzenberg et al. (Minzenberg et al., 2004) did not examine potential confounding variables, thus suggesting that the Pharmacological and Clinical Indexes may have been artifactually related to cognition through symptom intensity or other confounding variables.

The eight anticholinergic burden scales were more valid risk factors of cognitive impairment than antipsychotic polypharmacy. However, the eight scales did not exhibit a more significant association with cognitive impairment than CPZeq, lorazepam equivalents, or the number of psychotropic drugs, which were significantly associated with cognitive impairment, as reported in previous studies (Chakos et al., 2006; Ballesteros et al., 2018; Savić et al., 2021). These findings diverge from prior studies that emphasized the significant association between anticholinergic burden and cognitive performance, whereas CPZeq did not show a similar association (Ang et al., 2017; Eum et al., 2017; Ballesteros et al., 2018; Cuesta et al., 2022). However, previous research predominantly focused on validating cumulative anticholinergic burden without comparing it with the number of psychotropic drugs, despite the evident strong collinearity between these measures. Yet, our results show that cumulative anticholinergic burden and the number of psychotropic drugs are both valid risk factors for cognitive impairment, suggesting that the iatrogenic risks of cognitive impairment may arise from the accumulation of medications rather than the use of specific anticholinergic treatments. Besides, our study supports the validity of several tools beyond their original purposes. For instance, the eight anticholinergic burden scales can be used to assess the iatrogenic risks of cognitive impairment and, at the same time, evaluate the risks of other anticholinergic peripheral and central side effects, such as sedation, constipation, falls or delirium. Additionally, our results advocate for the use of the number of psychotropic drugs to assess the risk of iatrogenic cognitive impairment in SZ in clinical settings, as it may be easier and faster to compute than drug equivalents or anticholinergic burden scales.

We identified a significant association between the number of psychotropic drugs and worse performance in executive function and reasoning for people with SZ. Although the literature extensively covers antipsychotic polypharmacy (Élie et al., 2010), only a limited number of studies have specifically addressed the association between psychotropic polypharmacy and cognition in SZ (Chakos et al., 2006). The co-prescription of antipsychotics and other psychotropic drugs appears to have a varying effect on cognitive performance, depending on the specific substance used (Chakos et al., 2006; Ballon and Stroup, 2013), and does not ensure better outcomes (Glick et al., 2006; Längle et al., 2012). A review reported that approximately 50% of patients taking antipsychotics are comedicated (Möller et al., 2014). In addition, the number of patients with schizophrenia using at least four different medications increased, while the number of patients receiving monotherapy decreased, between 1994 and 2009 (Möller et al., 2014). Our results shed light on the common issue of psychotropic polypharmacy in psychiatry and are aligned with the recommendation of Zink et al. (Zink et al., 2010) to consider complementing antipsychotic medication with cognitive remediation, when feasible, to mitigate additional iatrogenic adverse effects.

Our study also highlights other factors associated with cognitive impairment. First, the residual association between cognition and medication was weak, thus suggesting that cognitive performance was mainly explained by non-iatrogenic factors. Among such factors, we observed a statistically significant association between the PANSS negative score and a decrease in global cognition, consistent with the findings of previous studies (Harvey et al., 2006; Ventura et al., 2009). Although previous studies did not identify any significant association between CGI-S values and cognitive functioning, our analysis showed CGI-S values to be significantly and moderately associated with cognition (Desmarais et al., 2014; Pinna et al., 2015). This suggests that treatment in psychiatry plays a minor role in exacerbating cognitive impairment compared to symptoms. However, we did identify a significant association between treatment and cognition, highlighting areas where current practices could be improved.

Our findings could significantly impact clinical decision making and outcomes in SZ. First, when a patient with SZ receives additional psychotropic medication, clinicians could plan a quick cognitive evaluation or use the cognitive dimension of the PANSS questionnaire in the coming weeks to anticipate and estimate the potential effect of the increased number of psychotropic drugs. Our results indicate that cognitive surveillance should pertain to any psychotropic medication and not only antipsychotics. Then, if a neuropsychological evaluation detects a cognitive deficit, reducing the number of psychotropic drugs could mitigate the iatrogenic burden on executive function and reasoning. To put the results in perspective, the strength of the association between the number of psychotropic drugs and cognitive performance corresponded to half that of the association between symptom severity and cognitive performance.

Our study was limited by its cross-sectional design. Despite our efforts to control for variables such as the history of psychosis, the subtypes of schizophrenia, and symptom severity, the interpretation of our results could be influenced by treatment indications. Patients with cognitive impairment might have received more medications than those without cognitive impairment due to the heightened severity of their symptoms or more frequent hospitalization (Ilzarbe and Vieta, 2023). Besides, only outpatients were included in the study, which may limit the generalizability of the results. Additionally, our sample was not big enough to encompass certain types of medications, such as tricyclic antidepressants, which could have provided valuable insights into their potential roles as risk factors for cognitive impairment in SZ (Podewils and Lyketsos, 2002).

Overall, our study confirms the significant association between anticholinergic burden scales and cognitive impairment in SZ. We identified eight valid scales to assess the risks of cognitive impairment, along with the number of psychotropic drugs and drug dose equivalents. Following the principle of parsimony, the number of psychotropic drugs can be recommended as an estimate of the risk of iatrogenic cognitive impairment in clinical or research applications, while the use of selected anticholinergic burden scales could be justified when additional hypotheses lead to a more specific investigation of the anticholinergic mechanisms.

## Data Availability

The data analyzed in this study is subject to the following licenses/restrictions: Due to ethical and legal restrictions, data involving clinical participants cannot be made publicly available. All relevant data are available upon request to the Foundation FondaMental for researchers who meet the criteria for access to confidential data. Requests to access these datasets should be directed to face@fondation-fondamental.org.

## Group members of the FACE-SZ (FondaMental Academic Centers of Expertise—Schizophrenia) Group

FACE-SZ Clinical Coordinating Center (Fondation FondaMental): F. Berna, E. Haffen, M. Leboyer, PM Llorca, and F. Schürhoff; FACE-SZ Data Coordinating Center (Fondation FondaMental): V. Barteau, S. Bensalem, O. Godin, H. Laouamri, and K. Souryis. AP-HP, INSERM U955, Translational Psychiatry Team, DHU Pe-PSY, Centre Expert Schizophrénie, Pôle de Psychiatrie et d'Addictologie des Hôpitaux Universitaires Henri Mondor, Paris Est University, 40 rue de Mesly, Créteil, France: E. Bourguignon, M. Leboyer, B. Pignon, J. Petrucci, F. Schürhoff, A. Szöke, and G. Wahiche; Department of Adult Psychiatry, Charles Perrens Hospital, Bordeaux, France; Laboratory of Nutrition and Integrative Neurobiology (UMR INRA 1286), University of Bordeaux, France: B. Aouizerate; Department of Adult Psychiatry, Charles Perrens Hospital, Bordeaux; University of Bordeaux, CNRS UMR 5287-INCIA, Bordeaux, France: A. Deloge, D. Misdrahi, and E. Vilà; CHU Clermont-Ferrand, Department of Psychiatry (service de psychiatrie B), University of Clermont Auvergne, Clermont-Ferrand, France: O. Blanc, I. Chéreau, H. Denizot, RM. Honciuc, D. Lacelle, PM. Llorca, and S. Pires; AP-HP, Department of Psychiatry, Louis Mourier Hospital, Colombes, Inserm UMR1266, Institute of Psychiatry and Neurosciences of Paris, University Paris Descartes, Université Paris Diderot, Sorbonne Paris Cité, Faculté de médecine, France: C. Dubertret, J. Mallet, and C. Portalier; Psychosocial Rehabilitation Reference Center, Alpes Isère Hospital, Grenoble, France: J. Dubreucq, C. Fluttaz, F. Gabayet, and C. Roman; University Claude Bernard Lyon 1, Le Vinatier Hospital Pole Est BP 300 39–95 bd Pinel–69678 Bron Cedex, France: G. Chesnoy-Servanin, T. D'Amato, JM. Dorey, R. Rey, and A. Vehier; Department of Psychiatry (AP-HM), Sainte-Marguerite University Hospital, Marseille, France: C. Lançon, C. Faget, E. Metairie, P. Peri, and F. Vaillant; AP-HM, la Conception Hospital, Aix-Marseille Univ, School of medicine–La Timone Medical Campus, EA 3279: CEReSS–Health Service Research: L. Boyer and G. Fond; Strasbourg University Hospital, University of Strasbourg, INSERM U1114, Federation of Translational Psychiatry, Strasbourg, France: F. Berna, P. Vidailhet, and A. Zinetti-Bertschy; University Department of Adult Psychiatry, La Colombiere Hospital, CHU Montpellier, University of Montpellier 1, Inserm 1061, Montpellier, France: D. Capdevielle and H. Yazbek; Versailles Hospital, Department of Adult Psychiatry and Addictology, Centre Hospitalier de Versailles, 177 rue de Versailles, 78157 Le Chesnay, France; DisAP-DevPsy-CESP, INSERM UMR1018, University of Paris-Saclay, University of Versailles Saint-Quentin-En-Yvelines 94807, Villejuif, France: S. Esselin, M. Jarroir, C. Passerieux, and M. Urbach.
