# Supplementary material for: Comparative analysis of anticholinergic burden scales to explain iatrogenic cognitive impairment in schizophrenia: results from the multicenter FACE-SZ cohort
Source: Front Pharmacol. 2024 Jun 12;15:1403093. doi: 10.3389/fphar.2024.1403093 (PMC11200119; doi:10.3389/fphar.2024.1403093)
Supplement: Supplementary file 1 [file Table1.PDF]

Supplementary Table 1. Pairwise correlations between the score in the Anticholinergic Cognitive Burden scale and the variables screened as potential covariates.

Variable	*Spearman rho coefficient*	*p-value*
Sex	-0.05	0.207
**Age**	0.10	**0.004**
Education level	-0.05	0.219
**CGI-S**	0.12	**0.001**
**PANSS positive score**	0.08	**0.026**
**PANSS negative score**	0.05	**0.132**
**Calgary**	0.14	**<.001**
Subtype of schizophrenia spectrum disorder	0.02	0.485
**Number of previous psychotic episodes**	0.16	**<.001**
**Number of hospitalization**	0.19	**<.001**

PANSS: the Positive And Negative Syndrome Scale for schizophrenia
Calgary: the Calgary Depression Rating Scale for Schizophrenia
CGI: Clinical Global Impression - Severity scale
