# Supplementary material for: Comparative analysis of anticholinergic burden scales to explain iatrogenic cognitive impairment in schizophrenia: results from the multicenter FACE-SZ cohort
Source: Front Pharmacol. 2024 Jun 12;15:1403093. doi: 10.3389/fphar.2024.1403093 (PMC11200119; doi:10.3389/fphar.2024.1403093)
Supplement: Supplementary file 2 [file Table2.PDF]

Supplementary Table 2. Results of the multiple linear regression models with the global cognition score as dependent variable. Significant associations (*p* < 0.05) are in bold. AAS: Anticholinergic Activity Scale, ABC: Anticholinergic Burden Classification, ACB: Anticholinergic Cognitive Burden scale, adj R2: adjusted R-squared, ADS: Anticholinergic Drug Scale, AEC: Anticholinergic Effect on Cognition, AIS: Anticholinergic Impregnation scale, ALS: Anticholinergic Load Scale, ARS: Anticholinergic Risk Scale, ATS: Anticholinergic Toxicity scale, BADS: Brazilian Anticholinergic Drug Scale, CALS: CRIDECO Anticholinergic Load Scale, Chew: Chew's Scale, CI: Clinical Index, DBI-WHO: Drug Burden Index WHO version, DRS: Delirogenic Risk Scale, Duran: Durán's scale, fmi: fraction of missing information, GACB: German Anticholinergic Burden scale, KABS: Korean Anticholinergic Burden Scale, mACB1: first modified Anticholinergic Cognitive Burden Scale, mACB2: second modified Anticholinergic Cognitive Burden Scale, MARANTE: Muscarinic Acetylcholinergic Receptor ANTagonist Exposure Scale, mARS: modified Anticholinergic Risk Scale, Peters: Peters's scale, PI: Pharmacological index, Salahudeen: Salahudeen's scale, Summers: Summers's scale.

	AAS (sum)[1]				AAS (max)[2]			
Var	Beta (95% CI)	p-value	Std Beta	fmi	Beta (95% CI)	p-value	Std Beta	fmi
AAS	-0.02 (-0.05: 0)	0.186	-0.06	0.30	-0.02 (-0.05:0.01)	0.333	-0.05	0.26
Age	0 (-0.01: 0)	0.664	-0.01	0.13	0 (-0.01:0)	0.708	-0.01	0.13
Calgary	0.01 (0: 0.02)	0.245	0.04	0.17	0.01 (0:0.02)	0.27	0.04	0.12
PANSS Positive score	0 (-0.01: 0)	0.353	-0.04	0.19	0 (-0.01:0.01)	0.347	-0.04	0.21
PANSS negative score	**-0.02 (-0.02: -0.01)**	**<.001**	-0.18	0.16	**-0.02 (-0.02:-0.01)**	**<.001**	-0.18	0.18
Number of hospitalization	-0.01 (-0.03: 0.01)	0.21	-0.06	0.25	-0.01 (-0.03:0)	0.14	-0.07	0.25
History of psychosis	-0.01 (-0.03: 0.01)	0.334	-0.04	0.26	-0.01 (-0.02:0.01)	0.356	-0.04	0.21
CGI	**-0.12 (-0.17: -0.06)**	**<.001**	-0.19	0.19	**-0.12 (-0.17:-0.06)**	**<.001**	-0.19	0.16

[1] $R2 = 0.136$; adj $R2 = 0.128$; AIC = 1660

[2] $R2 = 0.134$; adj $R2 = 0.125$; AIC = 1661

	ABC (sum)[1]				ABC (max)[2]			
Var	Beta (95% CI)	p-value	Std Beta	fmi	Beta (95% CI)	p-value	Std Beta	fmi
ABC	-0.02 (-0.05: 0.02)	0.319	-0.04	0.24	-0.01 (-0.05:0.03)	0.566	-0.02	0.29
Age	0 (-0.01: 0)	0.726	-0.01	0.14	0 (-0.01:0)	0.701	-0.01	0.13
Calgary	0.01 (0: 0.02)	0.211	0.05	0.18	0.01 (0:0.02)	0.248	0.04	0.13
PANSS Positive score	0 (-0.01: 0)	0.312	-0.04	0.18	0 (-0.01:0)	0.321	-0.04	0.21
PANSS negative score	**-0.02 (-0.02: -0.01)**	**<.001**	-0.18	0.15	**-0.02 (-0.02:-0.01)**	**<.001**	-0.18	0.18
Number of hospitalization	-0.01 (-0.03: 0)	0.156	-0.07	0.27	-0.01 (-0.03:0)	0.115	-0.08	0.24
History of psychosis	-0.01 (-0.03: 0.01)	0.364	-0.04	0.27	-0.01 (-0.02:0.01)	0.375	-0.04	0.21
CGI	**-0.12 (-0.17: -0.06)**	**<.001**	-0.19	0.19	**-0.12 (-0.17:-0.06)**	**<.001**	-0.19	0.16

[1] $R2 = 0.135$; adj $R2 = 0.126$; AIC = 1661

[2] $R2 = 0.133$; adj $R2 = 0.125$; AIC = 1661

	ACB (sum)[1]			
Var	Beta (95% CI)	p-value	Std Beta	fmi
ACB	**-0.03 (-0.05: 0)**	**0.043**	-0.07	0.13
Age	0 (-0.01: 0)	0.706	-0.01	0.13
Calgary	0.01 (0: 0.02)	0.176	0.05	0.16
PANSS Positive score	0 (-0.01: 0)	0.336	-0.04	0.19
PANSS negative score	**-0.02 (-0.02: -0.01)**	**<.001**	-0.18	0.15
Number of hospitalization	-0.01 (-0.03: 0.01)	0.213	-0.06	0.26
History of psychosis	-0.01 (-0.03: 0.01)	0.377	-0.04	0.27
CGI	**-0.12 (-0.17: -0.06)**	**<.001**	-0.19	0.19

[1] R2 = 0.138; adj R2 = 0.13; AIC = 1658

	ADS (sum)[1]			
Var	Beta (95% CI)	p-value	Std Beta	fmi
ADS	-0.02 (-0.05: 0)	0.098	-0.06	0.18
Age	0 (-0.01: 0)	0.688	-0.01	0.13
Calgary	0.01 (0: 0.02)	0.197	0.05	0.16
PANSS Positive score	0 (-0.01: 0)	0.346	-0.04	0.19
PANSS negative score	**-0.02 (-0.02: -0.01)**	**<.001**	-0.18	0.15
Number of hospitalization	-0.01 (-0.03: 0.01)	0.221	-0.06	0.25
History of psychosis	-0.01 (-0.03: 0.01)	0.358	-0.04	0.26
CGI	**-0.12 (-0.17: -0.06)**	**<.001**	-0.19	0.19

[1] R2 = 0.137; adj R2 = 0.128; AIC = 1659

	AEC (sum)[1]				AEC (max)[2]			
Var	Beta (95% CI)	p-value	Std Beta	fmi	Beta (95% CI)	p-value	Std Beta	fmi
AEC	**-0.04 (-0.07: -0.01)**	**0.008**	-0.09	0.13	-0.04 (-0.09:0.01)	0.121	-0.06	0.23
Age	0 (-0.01: 0)	0.649	-0.02	0.14	0 (-0.01:0)	0.709	-0.01	0.13
Calgary	0.01 (0: 0.02)	0.173	0.05	0.17	0.01 (0:0.02)	0.229	0.04	0.13
PANSS Positive score	0 (-0.01: 0)	0.33	-0.04	0.19	0 (-0.01:0.01)	0.345	-0.04	0.22
PANSS negative score	**-0.02 (-0.02: -0.01)**	**<.001**	-0.18	0.15	**-0.02 (-0.02:-0.01)**	**<.001**	-0.18	0.18
Number of hospitalization	-0.01 (-0.03: 0.01)	0.251	-0.06	0.25	-0.01 (-0.03:0)	0.15	-0.07	0.25
History of psychosis	-0.01 (-0.03: 0.01)	0.35	-0.04	0.25	-0.01 (-0.02:0.01)	0.381	-0.04	0.21
CGI	**-0.12 (-0.17: -0.06)**	**<.001**	-0.19	0.19	**-0.12 (-0.17:-0.06)**	**<.001**	-0.19	0.17

[1] R2 = 0.141; adj R2 = 0.133; AIC = 1654

[2] R2 = 0.136; adj R2 = 0.127; AIC = 1659

	AIS (sum)[1]				AIS (max)[2]			
Var	Beta (95% CI)	p-value	Std Beta	fmi	Beta (95% CI)	p-value	Std Beta	fmi
AIS	**-0.03 (-0.05: -0.01)**	**0.008**	-0.10	0.19	-0.04 (-0.1:0.02)	0.176	-0.05	0.24
Age	0 (-0.01: 0)	0.769	-0.01	0.13	0 (-0.01:0)	0.733	-0.01	0.13
Calgary	0.01 (0: 0.02)	0.161	0.05	0.16	0.01 (0:0.02)	0.259	0.04	0.12
PANSS Positive score	-0.01 (-0.01: 0)	0.297	-0.04	0.19	0 (-0.01:0)	0.321	-0.04	0.22
PANSS negative score	**-0.02 (-0.02: -0.01)**	**<.001**	-0.18	0.15	**-0.02 (-0.02:-0.01)**	**<.001**	-0.18	0.18
Number of hospitalization	-0.01 (-0.03: 0.01)	0.247	-0.06	0.26	-0.01 (-0.03:0)	0.143	-0.07	0.25
History of psychosis	-0.01 (-0.02: 0.01)	0.463	-0.04	0.27	-0.01 (-0.02:0.01)	0.396	-0.04	0.20
CGI	**-0.11 (-0.17: -0.06)**	**<.001**	-0.18	0.19	**-0.11 (-0.17:-0.06)**	**<.001**	-0.19	0.17

[1] R2 = 0.142; adj R2 = 0.134; AIC = 1653

[2] R2 = 0.135; adj R2 = 0.127; AIC = 1659

	ALS (sum)[1]				ALS (max)[2]			
Var	Beta (95% CI)	p-value	Std Beta	fmi	Beta (95% CI)	p-value	Std Beta	fmi
ALS	-0.01 (-0.07: 0.04)	0.609	-0.02	0.34	-0.04 (-0.11:0.04)	0.354	-0.04	0.30
Age	0 (-0.01: 0)	0.669	-0.02	0.14	0 (-0.01:0)	0.701	-0.01	0.13
Calgary	0.01 (0: 0.02)	0.227	0.04	0.15	0.01 (0:0.02)	0.232	0.04	0.12
PANSS Positive score	0 (-0.01: 0)	0.326	-0.04	0.19	0 (-0.01:0)	0.319	-0.04	0.21
PANSS negative score	**-0.02 (-0.02: -0.01)**	**<.001**	-0.18	0.15	**-0.02 (-0.02:-0.01)**	**<.001**	-0.18	0.18
Number of hospitalization	-0.01 (-0.03: 0)	0.16	-0.07	0.26	-0.01 (-0.03:0)	0.11	-0.08	0.25
History of psychosis	-0.01 (-0.03: 0.01)	0.346	-0.05	0.26	-0.01 (-0.02:0.01)	0.421	-0.04	0.22
CGI	**-0.12 (-0.17: -0.06)**	**<.001**	-0.19	0.19	**-0.12 (-0.17:-0.06)**	**<.001**	-0.19	0.17

[1] R2 = 0.134; adj R2 = 0.126; AIC = 1662

[2] R2 = 0.134; adj R2 = 0.126; AIC = 1660

	ARS (sum)[1]				ARS (max)[2]			
Var	Beta (95% CI)	p-value	Std Beta	fmi	Beta (95% CI)	p-value	Std Beta	fmi
ARS	-0.03 (-0.08: 0.02)	0.186	-0.05	0.30	-0.03 (-0.09:0.03)	0.333	-0.04	0.26
Age	0 (-0.01: 0)	0.664	-0.02	0.13	0 (-0.01:0)	0.708	-0.01	0.13
Calgary	0.01 (0: 0.02)	0.245	0.04	0.17	0.01 (-0.01:0.02)	0.27	0.04	0.12
PANSS Positive score	0 (-0.01: 0.01)	0.353	-0.04	0.19	0 (-0.01:0.01)	0.347	-0.04	0.21
PANSS negative score	**-0.02 (-0.02: -0.01)**	**<.001**	-0.18	0.16	**-0.02 (-0.02:-0.01)**	**<.001**	-0.18	0.18
Number of hospitalization	-0.01 (-0.03: 0.01)	0.21	-0.06	0.25	-0.01 (-0.03:0)	0.14	-0.07	0.25
History of psychosis	-0.01 (-0.03: 0.01)	0.334	-0.05	0.26	-0.01 (-0.02:0.01)	0.356	-0.04	0.21
CGI	**-0.12 (-0.17: -0.06)**	**<.001**	-0.19	0.19	**-0.12 (-0.17:-0.06)**	**<.001**	-0.19	0.16

[1] R2 = 0.136; adj R2 = 0.128; AIC = 1660

[2] R2 = 0.134; adj R2 = 0.125; AIC = 1661

	ATS (sum)[1]			
Var	Beta (95% CI)	p-value	Std Beta	fmi
ATS	-0.01 (-0.02: 0.01)	0.537	-0.02	0.21
Age	0 (-0.01: 0)	0.658	-0.02	0.14
Calgary	0.01 (0: 0.02)	0.234	0.04	0.16
PANSS Positive score	0 (-0.01: 0)	0.335	-0.04	0.19
PANSS negative score	**-0.02 (-0.02: -0.01)**	**<.001**	-0.18	0.16
Number of hospitalization	-0.01 (-0.03: 0)	0.161	-0.07	0.26
History of psychosis	-0.01 (-0.03: 0.01)	0.327	-0.05	0.26
CGI	**-0.12 (-0.17: -0.06)**	**<.001**	-0.19	0.19

[1] R2 = 0.134; adj R2 = 0.125; AIC = 1662

	BADS (sum)[1]				BADS (max)[2]			
Var	Beta (95% CI)	p-value	Std Beta	fmi	Beta (95% CI)	p-value	Std Beta	fmi
BADS	-0.02 (-0.05: 0)	0.059	-0.07	0.13	-0.03 (-0.07:0.02)	0.209	-0.05	0.20
Age	0 (-0.01: 0)	0.747	-0.01	0.13	0 (-0.01:0)	0.747	-0.01	0.13
Calgary	0.01 (0: 0.02)	0.178	0.05	0.16	0.01 (0:0.02)	0.254	0.04	0.12
PANSS Positive score	0 (-0.01: 0)	0.335	-0.04	0.19	0 (-0.01:0)	0.337	-0.04	0.22
PANSS negative score	**-0.02 (-0.02: -0.01)**	**<.001**	-0.18	0.15	**-0.02 (-0.02:-0.01)**	**<.001**	-0.18	0.18
Number of hospitalization	-0.01 (-0.03: 0.01)	0.214	-0.06	0.26	-0.01 (-0.03:0)	0.138	-0.07	0.25
History of psychosis	-0.01 (-0.03: 0.01)	0.372	-0.04	0.26	-0.01 (-0.02:0.01)	0.368	-0.04	0.21
CGI	**-0.12 (-0.17: -0.06)**	**<.001**	-0.19	0.19	**-0.12 (-0.17:-0.06)**	**<.001**	-0.19	0.16

[1] R2 = 0.137; adj R2 = 0.129; AIC = 1658

[2] R2 = 0.134; adj R2 = 0.126; AIC = 1660

Var	CALS (sum)[1]				CALS (max)[2]			
	Beta (95% CI)	p-value	Std Beta	fmi	Beta (95% CI)	p-value	Std Beta	fmi
CALS	**-0.03 (-0.05: 0)**	**0.04**	-0.08	0.20	-0.03 (-0.09:0.04)	0.419	-0.03	0.33
Age	0 (-0.01: 0)	0.726	-0.01	0.13	0 (-0.01:0)	0.701	-0.01	0.13
Calgary	0.01 (0: 0.02)	0.176	0.05	0.16	0.01 (-0.01:0.02)	0.27	0.04	0.12
PANSS Positive score	0 (-0.01: 0)	0.31	-0.04	0.19	0 (-0.01:0)	0.33	-0.04	0.21
PANSS negative score	**-0.02 (-0.02: -0.01)**	**<.001**	-0.18	0.15	**-0.02 (-0.02:-0.01)**	**<.001**	-0.18	0.18
Number of hospitalization	-0.01 (-0.03: 0.01)	0.232	-0.06	0.26	-0.01 (-0.03:0)	0.133	-0.07	0.25
History of psychosis	-0.01 (-0.02: 0.01)	0.422	-0.04	0.27	-0.01 (-0.02:0.01)	0.381	-0.04	0.20
CGI	**-0.11 (-0.17: -0.06)**	**<.001**	-0.18	0.19	**-0.12 (-0.17:-0.06)**	**<.001**	-0.19	0.16

[1] R^2 = 0.139; adj R^2 = 0.13; AIC = 1657

[2] R^2 = 0.134; adj R^2 = 0.125; AIC = 1661

Var	Chew (sum)[1]				Chew (max)[2]			
	Beta (95% CI)	p-value	Std Beta	fmi	Beta (95% CI)	p-value	Std Beta	fmi
Chew	-0.03 (-0.07: 0.01)	0.135	-0.06	0.20	-0.03 (-0.07:0.02)	0.273	-0.04	0.24
Age	0 (-0.01: 0)	0.645	-0.02	0.14	0 (-0.01:0)	0.693	-0.01	0.12
Calgary	0.01 (0: 0.02)	0.242	0.04	0.16	0.01 (-0.01:0.02)	0.272	0.04	0.12
PANSS Positive score	0 (-0.01: 0)	0.332	-0.04	0.19	0 (-0.01:0.01)	0.353	-0.04	0.22
PANSS negative score	**-0.02 (-0.03: -0.01)**	**<.001**	-0.18	0.15	**-0.02 (-0.02:-0.01)**	**<.001**	-0.18	0.18
Number of hospitalization	-0.01 (-0.03: 0.01)	0.217	-0.06	0.25	-0.01 (-0.03:0)	0.151	-0.07	0.25
History of psychosis	-0.01 (-0.03: 0.01)	0.34	-0.05	0.26	-0.01 (-0.02:0.01)	0.361	-0.04	0.21
CGI	**-0.12 (-0.17: -0.06)**	**<.001**	-0.19	0.19	**-0.12 (-0.17:-0.06)**	**<.001**	-0.19	0.16

[1] R^2 = 0.136; adj R^2 = 0.128; AIC = 1659

[2] R^2 = 0.134; adj R^2 = 0.126; AIC = 1660

	CI (sum)[1]				CI (max)[2]			
Var	Beta (95% CI)	p-value	Std Beta	fmi	Beta (95% CI)	p-value	Std Beta	fmi
CI	-0.03 (-0.1: 0.04)	0.427	-0.03	0.28	-0.02 (-0.1:0.05)	0.566	-0.02	0.17
Age	0 (-0.01: 0)	0.629	-0.02	0.13	0 (-0.01:0)	0.661	-0.02	0.13
Calgary	0.01 (0: 0.02)	0.251	0.04	0.16	0.01 (-0.01:0.02)	0.263	0.04	0.12
PANSS Positive score	0 (-0.01: 0.01)	0.364	-0.04	0.19	0 (-0.01:0.01)	0.353	-0.04	0.22
PANSS negative score	**-0.02 (-0.02: -0.01)**	**<.001**	-0.18	0.15	**-0.02 (-0.02:-0.01)**	**<.001**	-0.18	0.18
Number of hospitalization	-0.01 (-0.03: 0.01)	0.176	-0.07	0.26	-0.01 (-0.03:0)	0.125	-0.07	0.25
History of psychosis	-0.01 (-0.03: 0.01)	0.345	-0.05	0.26	-0.01 (-0.02:0.01)	0.376	-0.04	0.21
CGI	**-0.12 (-0.17: -0.06)**	**<.001**	-0.19	0.19	**-0.12 (-0.17:-0.06)**	**<.001**	-0.19	0.17

[1] $R2 = 0.134$; adj $R2 = 0.126$; AIC = 1661

[2] $R2 = 0.133$; adj $R2 = 0.124$; AIC = 1662

	CPZeq[1]			
Var	Beta (95% CI)	p-value	Std Beta	fmi
CPZeq	**-2e-04 (-3e-04: -1e-04)**	**0.005**	-0.11	0.28
Age	-0.001 (-0.007: 0.004)	0.635	-0.02	0.13
Calgary	0.006 (-0.006: 0.018)	0.31	0.04	0.18
PANSS Positive score	-0.004 (-0.013: 0.006)	0.412	-0.03	0.19
PANSS negative score	**-0.017 (-0.025: -0.01)**	**<.001**	-0.18	0.16
Number of hospitalization	-0.01 (-0.026: 0.007)	0.242	-0.06	0.28
History of psychosis	-0.007 (-0.024: 0.01)	0.421	-0.04	0.25
CGI	**-0.115 (-0.17: -0.06)**	**<.001**	-0.19	0.19

[1] $R2 = 0.145$; adj $R2 = 0.136$; AIC = 1651

	DBI-WHO (sum)[1]			
Var	Beta (95% CI)	p-value	Std Beta	fmi
DBI-WHO	-0.08 (-0.2: 0.04)	0.214	-0.05	0.24
Age	0 (-0.01: 0)	0.663	-0.02	0.13
Calgary	0.01 (0: 0.02)	0.179	0.05	0.15
PANSS Positive score	0 (-0.01: 0)	0.325	-0.04	0.19
PANSS negative score	**-0.02 (-0.02: -0.01)**	**<.001**	-0.18	0.15
Number of hospitalization	-0.01 (-0.03: 0)	0.168	-0.07	0.26
History of psychosis	-0.01 (-0.03: 0.01)	0.358	-0.04	0.27
CGI	**-0.12 (-0.17: -0.06)**	**<.001**	-0.19	0.19

[1] R2 = 0.135; adj R2 = 0.127; AIC = 1660

	DRS (sum)[1]				DRS (max)[2]			
Var	Beta (95% CI)	p-value	Std Beta	fmi	Beta (95% CI)	p-value	Std Beta	fmi
DRS	-0.02 (-0.04: 0)	0.066	-0.07	0.19	-0.02 (-0.05:0.01)	0.267	-0.04	0.21
Age	0 (-0.01: 0)	0.762	-0.01	0.13	0 (-0.01:0)	0.751	-0.01	0.13
Calgary	0.01 (0: 0.02)	0.164	0.05	0.16	0.01 (0:0.02)	0.231	0.04	0.13
PANSS Positive score	0 (-0.01: 0)	0.341	-0.04	0.19	0 (-0.01:0.01)	0.346	-0.04	0.22
PANSS negative score	**-0.02 (-0.02: -0.01)**	**<.001**	-0.18	0.15	**-0.02 (-0.02:-0.01)**	**<.001**	-0.18	0.17
Number of hospitalization	-0.01 (-0.03: 0.01)	0.211	-0.06	0.26	-0.01 (-0.03:0)	0.142	-0.07	0.25
History of psychosis	-0.01 (-0.03: 0.01)	0.379	-0.04	0.26	-0.01 (-0.02:0.01)	0.369	-0.04	0.21
CGI	**-0.12 (-0.17: -0.06)**	**<.001**	-0.19	0.19	**-0.12 (-0.17:-0.06)**	**<.001**	-0.19	0.16

[1] R2 = 0.138; adj R2 = 0.129; AIC = 1658

[2] R2 = 0.134; adj R2 = 0.126; AIC = 1660

	Duran (sum)[1]				Duran (max)[2]			
Var	Beta (95% CI)	p-value	Std Beta	fmi	Beta (95% CI)	p-value	Std Beta	fmi
Duran	**-0.03 (-0.05: -0.01)**	**0.013**	-0.09	0.21	-0.02 (-0.08:0.03)	0.37	-0.03	0.21
Age	0 (-0.01: 0)	0.803	-0.01	0.14	0 (-0.01:0)	0.737	-0.01	0.13
Calgary	0.01 (0: 0.02)	0.165	0.05	0.16	0.01 (0:0.02)	0.253	0.04	0.13
PANSS Positive score	0 (-0.01: 0)	0.336	-0.04	0.19	0 (-0.01:0)	0.339	-0.04	0.21
PANSS negative score	**-0.02 (-0.02: -0.01)**	**<.001**	-0.18	0.15	**-0.02 (-0.02:-0.01)**	**<.001**	-0.18	0.18
Number of hospitalization	-0.01 (-0.03: 0.01)	0.221	-0.06	0.27	-0.01 (-0.03:0)	0.139	-0.07	0.25
History of psychosis	-0.01 (-0.02: 0.01)	0.465	-0.04	0.28	-0.01 (-0.02:0.01)	0.381	-0.04	0.20
CGI	**-0.11 (-0.17: -0.06)**	**<.001**	-0.18	0.19	**-0.12 (-0.17:-0.06)**	**<.001**	-0.19	0.16

[1] R2 = 0.141; adj R2 = 0.133; AIC = 1654

[2] R2 = 0.134; adj R2 = 0.125; AIC = 1661

	GACB (sum)[1]				GACB (max)[2]			
Var	Beta (95% CI)	p-value	Std Beta	fmi	Beta (95% CI)	p-value	Std Beta	fmi
GACB	**-0.03 (-0.06: 0)**	**0.043**	-0.08	0.19	-0.03 (-0.09:0.03)	0.379	-0.03	0.25
Age	0 (-0.01: 0)	0.749	-0.01	0.13	0 (-0.01:0)	0.716	-0.01	0.13
Calgary	0.01 (0: 0.02)	0.158	0.05	0.16	0.01 (0:0.02)	0.251	0.04	0.12
PANSS Positive score	0 (-0.01: 0)	0.342	-0.04	0.19	0 (-0.01:0)	0.338	-0.04	0.22
PANSS negative score	**-0.02 (-0.02: -0.01)**	**<.001**	-0.18	0.15	**-0.02 (-0.02:-0.01)**	**<.001**	-0.18	0.18
Number of hospitalization	-0.01 (-0.03: 0.01)	0.21	-0.06	0.27	-0.01 (-0.03:0)	0.138	-0.07	0.25
History of psychosis	-0.01 (-0.02: 0.01)	0.429	-0.04	0.27	-0.01 (-0.02:0.01)	0.381	-0.04	0.21
CGI	**-0.12 (-0.17: -0.06)**	**<.001**	-0.19	0.19	**-0.12 (-0.17:-0.06)**	**<.001**	-0.19	0.16

[1] R2 = 0.138; adj R2 = 0.13; AIC = 1657

[2] R2 = 0.134; adj R2 = 0.125; AIC = 1661

	KABS (sum)[1]				KABS (max)[2]			
Var	Beta (95% CI)	p-value	Std Beta	fmi	Beta (95% CI)	p-value	Std Beta	fmi
KABS	**-0.03 (-0.06: 0)**	**0.035**	-0.08	0.16	-0.03 (-0.09:0.02)	0.228	-0.04	0.21
Age	0 (-0.01: 0)	0.752	-0.01	0.13	0 (-0.01:0)	0.728	-0.01	0.13
Calgary	0.01 (0: 0.02)	0.148	0.05	0.16	0.01 (0:0.02)	0.257	0.04	0.12
PANSS Positive score	0 (-0.01: 0)	0.327	-0.04	0.19	0 (-0.01:0)	0.335	-0.04	0.22
PANSS negative score	**-0.02 (-0.02: -0.01)**	**<.001**	-0.18	0.15	**-0.02 (-0.02:-0.01)**	**<.001**	-0.18	0.17
Number of hospitalization	-0.01 (-0.03: 0.01)	0.225	-0.06	0.26	-0.01 (-0.03:0)	0.14	-0.07	0.25
History of psychosis	-0.01 (-0.03: 0.01)	0.386	-0.04	0.27	-0.01 (-0.02:0.01)	0.353	-0.04	0.21
CGI	**-0.12 (-0.17: -0.06)**	**<.001**	-0.19	0.19	**-0.11 (-0.17:-0.06)**	**<.001**	-0.19	0.16

[1] $R2 = 0.139$; adj $R2 = 0.13$; $AIC = 1657$

[2] $R2 = 0.134$; adj $R2 = 0.126$; $AIC = 1660$

	LORAZeq[1]			
Var	Beta (95% CI)	p-value	Std Beta	fmi
LORAZeq	**-0.09 (-0.17: -0.01)**	**0.037**	-0.09	0.35
Age	0 (-0.01: 0)	0.795	-0.01	0.14
Calgary	0.01 (0: 0.02)	0.201	0.05	0.17
PANSS Positive score	0 (-0.01: 0.01)	0.398	-0.03	0.19
PANSS negative score	**-0.02 (-0.02: -0.01)**	**<.001**	-0.18	0.15
Number of hospitalization	-0.01 (-0.03: 0.01)	0.18	-0.07	0.27
History of psychosis	-0.01 (-0.03: 0.01)	0.402	-0.04	0.28
CGI	**-0.12 (-0.18: -0.07)**	**<.001**	-0.20	0.19

[1] $R2 = 0.14$; adj $R2 = 0.132$; $AIC = 1655$

Var	mACB1 (sum)[1]				mACB1 (max)[2]			
	Beta (95% CI)	p-value	Std Beta	fmi	Beta (95% CI)	p-value	Std Beta	fmi
mACB1	-0.02 (-0.05: 0.01)	0.152	-0.05	0.15	-0.02 (-0.07:0.03)	0.393	-0.03	0.25
Age	0 (-0.01: 0)	0.668	-0.02	0.13	0 (-0.01:0)	0.698	-0.01	0.13
Calgary	0.01 (0: 0.02)	0.21	0.05	0.17	0.01 (0:0.02)	0.259	0.04	0.12
PANSS Positive score	0 (-0.01: 0)	0.321	-0.04	0.19	0 (-0.01:0)	0.334	-0.04	0.22
PANSS negative score	**-0.02 (-0.02: -0.01)**	**<.001**	-0.18	0.15	**-0.02 (-0.02:-0.01)**	**<.001**	-0.18	0.18
Number of hospitalization	-0.01 (-0.03: 0.01)	0.182	-0.07	0.26	-0.01 (-0.03:0)	0.13	-0.07	0.25
History of psychosis	-0.01 (-0.03: 0.01)	0.352	-0.04	0.26	-0.01 (-0.02:0.01)	0.369	-0.04	0.21
CGI	**-0.12 (-0.17: -0.06)**	**<.001**	-0.19	0.19	**-0.12 (-0.17:-0.06)**	**<.001**	-0.19	0.17

[1] $R2 = 0.136$; adj $R2 = 0.127$; $AIC = 1660$

[2] $R2 = 0.134$; adj $R2 = 0.125$; $AIC = 1661$

Var	mACB2 (sum)[1]			
	Beta (95% CI)	p-value	Std Beta	fmi
mACB2	-0.02 (-0.04: 0.01)	0.122	-0.06	0.12
Age	0 (-0.01: 0)	0.712	-0.01	0.13
Calgary	0.01 (0: 0.02)	0.186	0.05	0.16
PANSS Positive score	0 (-0.01: 0)	0.334	-0.04	0.19
PANSS negative score	**-0.02 (-0.02: -0.01)**	**<.001**	-0.18	0.15
Number of hospitalization	-0.01 (-0.03: 0.01)	0.202	-0.06	0.26
History of psychosis	-0.01 (-0.03: 0.01)	0.375	-0.04	0.26
CGI	**-0.12 (-0.17: -0.06)**	**<.001**	-0.19	0.19

[1] $R2 = 0.136$; adj $R2 = 0.128$; $AIC = 1660$

	Marante (sum)[1]				Marante (max)[2]			
Var	Beta (95% CI)	p-value	Std Beta	fmi	Beta (95% CI)	p-value	Std Beta	fmi
Marante	-0.03 (-0.07: 0)	0.05	-0.08	0.25	-0.02 (-0.08:0.04)	0.539	-0.02	0.28
Age	0 (-0.01: 0)	0.812	-0.01	0.13	0 (-0.01:0)	0.72	-0.01	0.13
Calgary	0.01 (0: 0.02)	0.182	0.05	0.15	0.01 (0:0.02)	0.252	0.04	0.13
PANSS Positive score	0 (-0.01: 0)	0.337	-0.04	0.19	0 (-0.01:0)	0.334	-0.04	0.22
PANSS negative score	**-0.02 (-0.02: -0.01)**	**<.001**	-0.18	0.15	**-0.02 (-0.02:-0.01)**	**<.001**	-0.18	0.19
Number of hospitalization	-0.01 (-0.03: 0.01)	0.186	-0.07	0.27	-0.01 (-0.03:0)	0.124	-0.07	0.25
History of psychosis	-0.01 (-0.02: 0.01)	0.432	-0.04	0.27	-0.01 (-0.02:0.01)	0.368	-0.04	0.21
CGI	**-0.11 (-0.17: -0.06)**	**<.001**	-0.18	0.20	**-0.12 (-0.17:-0.06)**	**<.001**	-0.19	0.17

[1] R2 = 0.139; adj R2 = 0.13; AIC = 1657

[2] R2 = 0.133; adj R2 = 0.125; AIC = 1661

	mARS (sum)[1]				mARS (max)[2]			
Var	Beta (95% CI)	p-value	Std Beta	fmi	Beta (95% CI)	p-value	Std Beta	fmi
mARS	-0.04 (-0.08: 0.01)	0.152	-0.06	0.29	-0.03 (-0.09:0.03)	0.285	-0.04	0.27
Age	0 (-0.01: 0)	0.688	-0.01	0.14	0 (-0.01:0)	0.714	-0.01	0.13
Calgary	0.01 (0: 0.02)	0.227	0.04	0.17	0.01 (-0.01:0.02)	0.267	0.04	0.12
PANSS Positive score	0 (-0.01: 0.01)	0.355	-0.04	0.19	0 (-0.01:0.01)	0.351	-0.04	0.22
PANSS negative score	**-0.02 (-0.02: -0.01)**	**<.001**	-0.18	0.15	**-0.02 (-0.02:-0.01)**	**<.001**	-0.18	0.18
Number of hospitalization	-0.01 (-0.03: 0.01)	0.201	-0.06	0.25	-0.01 (-0.03:0)	0.144	-0.07	0.25
History of psychosis	-0.01 (-0.03: 0.01)	0.347	-0.05	0.26	-0.01 (-0.03:0.01)	0.353	-0.04	0.21
CGI	**-0.12 (-0.17: -0.06)**	**<.001**	-0.19	0.19	**-0.12 (-0.17:-0.06)**	**<.001**	-0.19	0.16

[1] R2 = 0.136; adj R2 = 0.128; AIC = 1659

[2] R2 = 0.134; adj R2 = 0.126; AIC = 1660

	Number of antipsychotics[1]			
Var	Beta (95% CI)	p-value	Std Beta	fmi
Number of antipsychotics	-0.08 (-0.17: 0.01)	0.069	-0.07	0.27
Age	0 (-0.01: 0)	0.636	-0.02	0.13
Calgary	0.01 (0: 0.02)	0.241	0.04	0.17
PANSS Positive score	0 (-0.01: 0)	0.326	-0.04	0.19
PANSS negative score	**-0.02 (-0.02: -0.01)**	**<.001**	-0.18	0.15
Number of hospitalization	-0.01 (-0.03: 0.01)	0.197	-0.06	0.26
History of psychosis	-0.01 (-0.03: 0.01)	0.38	-0.04	0.26
CGI	**-0.12 (-0.17: -0.06)**	**<.001**	-0.19	0.19

[1] R2 = 0.138; adj R2 = 0.13; AIC = 1658

	Number of psychotropic drugs[1]			
Var	Beta (95% CI)	p-value	Std Beta	fmi
Number of psychotropic drugs	**-0.05 (-0.09: -0.01)**	**0.016**	-0.10	0.28
Age	0 (-0.01: 0)	0.832	-0.01	0.14
Calgary	0.01 (0: 0.02)	0.094	0.06	0.15
PANSS Positive score	-0.01 (-0.01: 0)	0.285	-0.04	0.20
PANSS negative score	**-0.02 (-0.02: -0.01)**	**<.001**	-0.18	0.15
Number of hospitalization	-0.01 (-0.03: 0.01)	0.221	-0.06	0.26
History of psychosis	-0.01 (-0.02: 0.01)	0.459	-0.04	0.26
CGI	**-0.11 (-0.17: -0.06)**	**<.001**	-0.18	0.19

[1] R2 = 0.142; adj R2 = 0.133; AIC = 1654

	Peters (sum)[1]				Peters (max)[2]			
Var	Beta (95% CI)	p-value	Std Beta	fmi	Beta (95% CI)	p-value	Std Beta	fmi
Peters	-0.04 (-0.1: 0.03)	0.306	-0.04	0.33	-0.02 (-0.09:0.05)	0.535	-0.02	0.30
Age	0 (-0.01: 0)	0.65	-0.02	0.14	0 (-0.01:0)	0.675	-0.02	0.13
Calgary	0.01 (0: 0.02)	0.222	0.04	0.17	0.01 (0:0.02)	0.244	0.04	0.13
PANSS Positive score	0 (-0.01: 0.01)	0.353	-0.04	0.19	0 (-0.01:0.01)	0.35	-0.04	0.22
PANSS negative score	**-0.02 (-0.02: -0.01)**	**<.001**	-0.18	0.16	**-0.02 (-0.02:-0.01)**	**<.001**	-0.18	0.18
Number of hospitalization	-0.01 (-0.03: 0.01)	0.181	-0.07	0.26	-0.01 (-0.03:0)	0.127	-0.07	0.25
History of psychosis	-0.01 (-0.03: 0.01)	0.332	-0.05	0.26	-0.01 (-0.02:0.01)	0.364	-0.04	0.21
CGI	**-0.12 (-0.17: -0.06)**	**<.001**	-0.19	0.19	**-0.12 (-0.17:-0.06)**	**<.001**	-0.19	0.17

[1] R2 = 0.135; adj R2 = 0.127; AIC = 1660

[2] R2 = 0.133; adj R2 = 0.125; AIC = 1661

	PI (sum)[1]				PI (max)[2]			
Var	Beta (95% CI)	p-value	Std Beta	fmi	Beta (95% CI)	p-value	Std Beta	fmi
PI	-0.01 (-0.02: 0)	0.144	-0.05	0.19	-0.01 (-0.02:0)	0.177	-0.05	0.20
Age	0 (-0.01: 0)	0.624	-0.02	0.14	0 (-0.01:0)	0.657	-0.02	0.13
Calgary	0.01 (-0.01: 0.02)	0.283	0.04	0.16	0.01 (-0.01:0.02)	0.297	0.04	0.12
PANSS Positive score	0 (-0.01: 0.01)	0.361	-0.04	0.18	0 (-0.01:0.01)	0.368	-0.04	0.22
PANSS negative score	**-0.02 (-0.02: -0.01)**	**<.001**	-0.18	0.15	**-0.02 (-0.02:-0.01)**	**<.001**	-0.18	0.18
Number of hospitalization	-0.01 (-0.03: 0.01)	0.219	-0.06	0.26	-0.01 (-0.03:0)	0.16	-0.07	0.25
History of psychosis	-0.01 (-0.03: 0.01)	0.332	-0.05	0.25	-0.01 (-0.02:0.01)	0.373	-0.04	0.22
CGI	**-0.12 (-0.17: -0.06)**	**<.001**	-0.19	0.19	**-0.12 (-0.17:-0.06)**	**<.001**	-0.19	0.17

[1] R2 = 0.136; adj R2 = 0.128; AIC = 1660

[2] R2 = 0.135; adj R2 = 0.126; AIC = 1660

Var	Salahudeen (sum)[1]				Salahudeen (max)[2]			
	Beta (95% CI)	p-value	Std Beta	fmi	Beta (95% CI)	p-value	Std Beta	fmi
Salahudeen	**-0.03 (-0.06: 0)**	**0.024**	-0.09	0.20	-0.02 (-0.09:0.04)	0.466	-0.03	0.41
Age	0 (-0.01: 0)	0.734	-0.01	0.14	0 (-0.01:0)	0.703	-0.01	0.13
Calgary	0.01 (0: 0.02)	0.155	0.05	0.16	0.01 (0:0.02)	0.254	0.04	0.13
PANSS Positive score	0 (-0.01: 0)	0.307	-0.04	0.19	0 (-0.01:0)	0.326	-0.04	0.21
PANSS negative score	**-0.02 (-0.02: -0.01)**	**<.001**	-0.18	0.15	**-0.02 (-0.02:-0.01)**	**<.001**	-0.18	0.18
Number of hospitalization	-0.01 (-0.03: 0.01)	0.213	-0.06	0.26	-0.01 (-0.03:0)	0.127	-0.07	0.24
History of psychosis	-0.01 (-0.02: 0.01)	0.439	-0.04	0.27	-0.01 (-0.02:0.01)	0.382	-0.04	0.21
CGI	**-0.11 (-0.17: -0.06)**	**<.001**	-0.18	0.18	**-0.12 (-0.17:-0.06)**	**<.001**	-0.19	0.16

[1] R2 = 0.14; adj R2 = 0.132; AIC = 1656

[2] R2 = 0.134; adj R2 = 0.125; AIC = 1661

Var	Summers (sum)[1]				Summers (max)[2]			
	Beta (95% CI)	p-value	Std Beta	fmi	Beta (95% CI)	p-value	Std Beta	fmi
Summers	-0.02 (-0.04: 0)	0.131	-0.06	0.28	-0.02 (-0.04:0.01)	0.151	-0.06	0.29
Age	0 (-0.01: 0)	0.641	-0.02	0.14	0 (-0.01:0)	0.687	-0.01	0.13
Calgary	0.01 (0: 0.02)	0.207	0.05	0.17	0.01 (0:0.02)	0.209	0.04	0.13
PANSS Positive score	0 (-0.01: 0.01)	0.375	-0.04	0.19	0 (-0.01:0.01)	0.377	-0.04	0.23
PANSS negative score	**-0.02 (-0.02: -0.01)**	**<.001**	-0.18	0.15	**-0.02 (-0.02:-0.01)**	**<.001**	-0.18	0.18
Number of hospitalization	-0.01 (-0.03: 0.01)	0.192	-0.06	0.26	-0.01 (-0.03:0)	0.146	-0.07	0.25
History of psychosis	-0.01 (-0.03: 0.01)	0.325	-0.05	0.26	-0.01 (-0.02:0.01)	0.356	-0.04	0.20
CGI	**-0.12 (-0.17: -0.06)**	**<.001**	-0.19	0.19	**-0.12 (-0.17:-0.06)**	**<.001**	-0.19	0.17

[1] R2 = 0.137; adj R2 = 0.128; AIC = 1659

[2] R2 = 0.136; adj R2 = 0.127; AIC = 1659
