# Supplementary material for: Comparative analysis of anticholinergic burden scales to explain iatrogenic cognitive impairment in schizophrenia: results from the multicenter FACE-SZ cohort
Source: Front Pharmacol. 2024 Jun 12;15:1403093. doi: 10.3389/fphar.2024.1403093 (PMC11200119; doi:10.3389/fphar.2024.1403093)
Supplement: Supplementary file 3 [file Image2.PDF]

Supplementary Figure 2. Patients' scores in each scale (n = 839). Dark grey: patients with a high anticholinergic burden score in the scale (thresholds used to define high anticholinergic burden scores are explained in supplementary information SM3). Grey: patients with a low anticholinergic burden score in the scale. Light grey: patients with a zero score in the scale. White: patients with missing data for treatment and therefore no score in the scales. The dotted line corresponds to the number of patients with a treatment in our sample (n = 666).

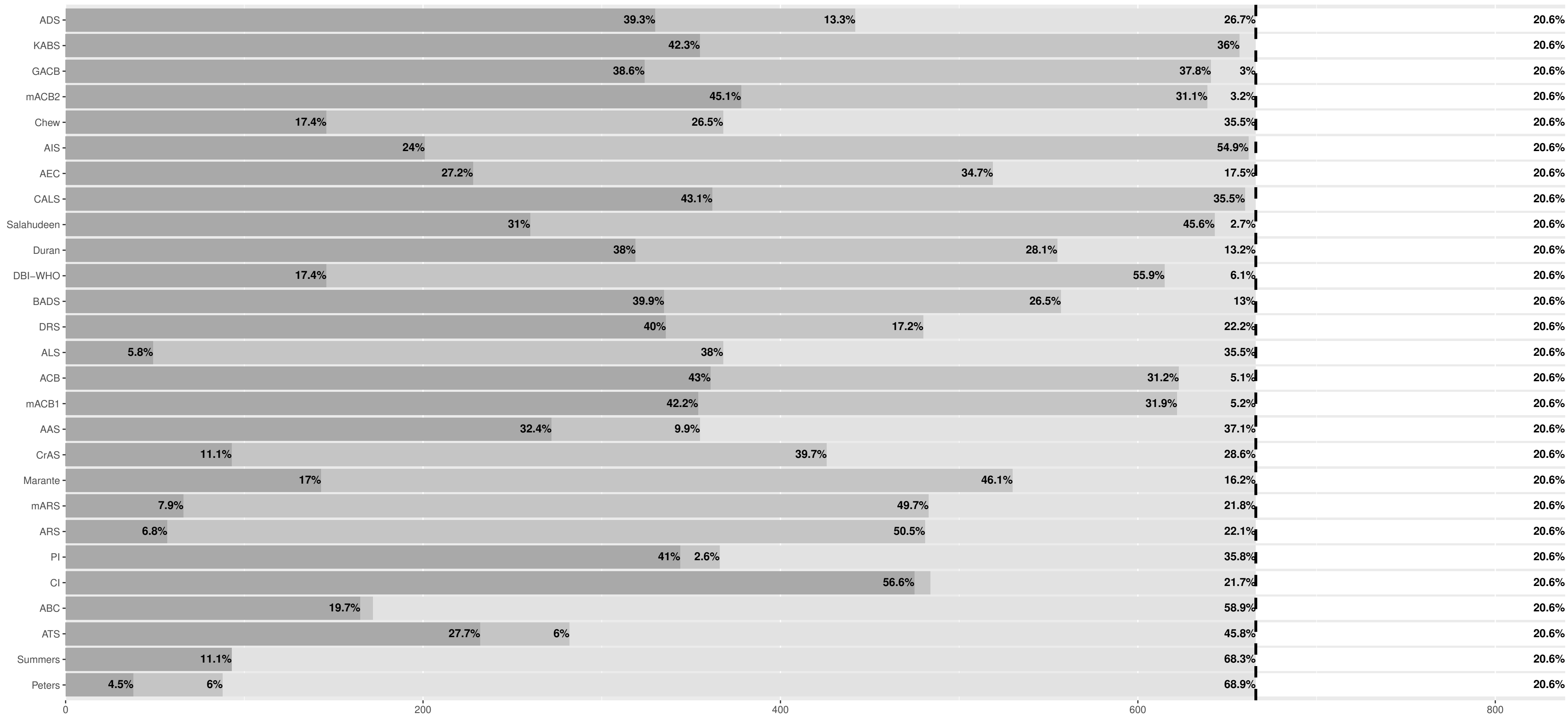
ADS 39.3% 13.3% 26.7% 20.6%
KABS 42.3% 36% 20.6%
GACB 38.6% 37.8% 3% 20.6%
mACB2 45.1% 31.1% 3.2% 20.6%
Chew 17.4% 26.5% 35.5% 20.6%
AIS 24% 54.9% 20.6%
AEC 27.2% 34.7% 17.5% 20.6%
CALS 43.1% 35.5% 20.6%
Salahudeen 31% 45.6% 2.7% 20.6%
Duran 38% 28.1% 13.2% 20.6%
DBI−WHO 17.4% 55.9% 6.1% 20.6%
BADS 39.9% 26.5% 13% 20.6%
DRS 40% 17.2% 22.2% 20.6%
ALS 5.8% 38% 35.5% 20.6%
ACB 43% 31.2% 5.1% 20.6%
mACB1 42.2% 31.9% 5.2% 20.6%
AAS 32.4% 9.9% 37.1% 20.6%
CrAS 11.1% 39.7% 28.6% 20.6%
Marante 17% 46.1% 16.2% 20.6%
mARS 7.9% 49.7% 21.8% 20.6%
ARS 6.8% 50.5% 22.1% 20.6%
PI 41% 2.6% 35.8% 20.6%
CI 56.6% 21.7% 20.6%
ABC 19.7% 58.9% 20.6%
ATS 27.7% 6% 45.8% 20.6%
Summers 11.1% 68.3% 20.6%
Peters 4.5% 6% 68.9% 20.6%
0
200
400
600
800
