# Supplementary material for: Comparative analysis of anticholinergic burden scales to explain iatrogenic cognitive impairment in schizophrenia: results from the multicenter FACE-SZ cohort
Source: Front Pharmacol. 2024 Jun 12;15:1403093. doi: 10.3389/fphar.2024.1403093 (PMC11200119; doi:10.3389/fphar.2024.1403093)
Supplement: Supplementary file 4 [file DataSheet1.DOCX]

# **Comparative analysis of anticholinergic burden scales to explain iatrogenic cognitive impairment in schizophrenia: results from the multicenter FACE-SZ cohort**

**Supporting information**

### **Supplementary material (SM1): selection of anticholinergic burden scales based on literature review**

We conducted a comprehensive literature review in November 2022 using various databases, including Google Scholar, PubMed, and Cochrane, with specific keywords related to anticholinergic properties and measurement scales.

*Search query:* ("anticholinergic"[Title/Abstract] OR "cholinergic antagonists"[Title/Abstract] OR "antimuscarinic"[Title/Abstract] OR "muscarinic antagonists" [Title/Abstract] OR "atropinic"[Title/Abstract]) AND ("scale"[Title/Abstract] OR "measure"[Title/Abstract] OR "tool"[Title/Abstract] OR "score"[Title/Abstract])

We identified a total of 36 scales that were designed to assess the anticholinergic properties of treatments. Information about the scales is summarized in the table below (excluded scales are indicated in grey).

| **Date** | **Scale (short name)** | **Exclusion/Inclusion** | **Country** | **Number of drugs scored** | **Type** | **Dose dependent** | **Method of design** |
| --- | --- | --- | --- | --- | --- | --- | --- |
| 1978 | **Summers’s scale** [**(1)**](https://www.zotero.org/google-docs/?K1Wzx3) **(Summer)** | Included (original version) | USA | 67 | numerical, discrete, from 1 to 3 | Yes (daily effective dosage) | Evaluation of delirium incidence of the drugs taken by 84 patients |
| 1989 | **Peters’s Scale** [**(2)**](https://www.zotero.org/google-docs/?6BfAUj) **(Peters)** | Included and recoded:  We coded “low” anticholinergic activity as 1, “intermediate” as 2, and “high” as 3. | USA | 21 | categorical (“low/intermediate/high anticholinergic activity”) | No | Literature review of dissociation constants for muscarinic receptors |
| 1992 | **Tune's list** [**(3)**](https://www.zotero.org/google-docs/?G2sMiC) | Excluded because psychotropic drugs were excluded in the design of the scale. | USA | 25 | numerical, continuous (atropine equivalents) | Yes | Radioreceptor assay of the 25 most prescribed drugs in elderly patients |
| 2001 | **Clinician-Rated Anticholinergic Score** [**(4)**](https://www.zotero.org/google-docs/?1Ya9bG) **(CR-ACh)** | Excluded in favor of a revised and more recent version, the CrAS. | Canada | 340 | numerical, discrete, from 0 to 3 | No | Expert committee (3 geriatric psychiatrists) |
| 2002 | **modified CR-ACh** [**(5)**](https://www.zotero.org/google-docs/?OgQN4v) **(CR-ACh-mod)** | Excluded in favor of a revised and more recent version, the ADS. | USA | 520 | numerical, discrete, from 0 to 3 | No | Expert committee consensus (3 psychiatric pharmacists) |
| 2002 | **Aizenberg’s scale** [**(6)**](https://www.zotero.org/google-docs/?5X9kW0) | Excluded because unavailable upon request to the authors. | Israël |  |  |  | Expert committee |
| 2004 | **Clinical index** [**(7)**](https://www.zotero.org/google-docs/?ezpaYX) **(CI)** | Included | USA | 28 | numerical, continuous (mg/mL, benztropine equivalents) | Yes | Expert committee (10 psychiatrists) made a decision based on their patients’ complaints of dry mouth, blurred vision, and constipation |
|  | **Pharmacological index** [**(7)**](https://www.zotero.org/google-docs/?kFhG3H) **(PI)** | Included |  |  |  |  | Expert committee (10 psychiatrists) made a decision based on a literature review of relative affinities of the drug for the muscarinic receptor |
| 2006 | **Anticholinergic Drug Scale** [**(8)**](https://www.zotero.org/google-docs/?68lycB) **(ADS)** | Included (its updated version released by the same authors in 2021) | USA | 520 in the original version, 1058 in the updated version | numerical, discrete, from 0 to 3 | No | Expert committee consensus (3 psychiatric pharmacists) |
| 2006 | **Anticholinergic Burden Classification** [**(9)**](https://www.zotero.org/google-docs/?9yhve9) **(ABC)** | Included (original version) | France | 27 | numerical, discrete, from 1 to 3 | No | Expert committee (1 pharmacologist, 1 physician, 1 biologist) |
| 2007 | **Cancelli's anticholinergic burden score** [**(10)**](https://www.zotero.org/google-docs/?0JJR1S) | Excluded because antipsychotics were excluded in the design of the scale. | Italy | 17 | numerical, discrete, from 0 to 3 | No | Expert committee (3 neurologists) |
| 2007 | **Drug Burden Index** [**(11)**](https://www.zotero.org/google-docs/?DhaINy) **(DBI)** | Excluded in favor of a more universal and recent version, DBI-WHO | USA Australia | NA (formula) | numerical, continuous | Yes | Literature review to design the formula |
| 2008 | **Clinician-Rated Anticholinergic score** [**(12)**](https://www.zotero.org/google-docs/?WvmX1X) **(CrAS)** | Included (original version) | Canada | 624 (only the 60 drugs with anticholinergic activity were given) | numerical, discrete, from 1 to 3 | No | Expert committee (3 geriatricians) |
| 2008 | **Cao’s score** [**(13)**](https://www.zotero.org/google-docs/?J9YfQf) **(similar to DBI)** | Excluded in favor of a more universal and recent version, DBI-WHO | USA Australia | NA (formula) | numerical, continuous | Yes | Literature review to design the formula |
| 2008 | **Anticholinergic Risk Scale** [**(14)**](https://www.zotero.org/google-docs/?Q9I1Gb) **(ARS)** | Included | USA | 49 | numerical, discrete, from 1 to 3 | No | Expert committee (1 geriatrician, 2 pharmacists) |
| 2008 | **Anticholinergic Cognitive Burden scale** [**(15)**](https://www.zotero.org/google-docs/?S1IF0d) **(ACB)** | Included (its updated version released by the same authors in 2012) | USA | 88 in the original version, 106 in the update | numerical, discrete, from 0 to 3 | No | Expert committee (geriatricians, geriatric pharmacists, psychiatrists and nurses, general physicians, aging brain researcher) |
| 2008 | **Chew’s Scale** [**(16)**](https://www.zotero.org/google-docs/?BepdK1) **(Chew)** | Included and recoded:  We used the same scoring used by Lisibach et al^21^ and coded “0” as 0, “+” as 1, “++” as 2, and “+++” as 3. In addition, we coded “+/0” as 0.5. | USA | 107 | categorical (“0”, “+/0”, “+”, “++”, “+++”) | No | Radioreceptor assay measurements of serum anticholinergic activity |
| 2010 | **Anticholinergic Activity Scale** [**(17)**](https://www.zotero.org/google-docs/?3jfL5E) **(AAS)** | Included | Norway | 99 | numerical, discrete, from 0 to 4 | No | Expert committee (2 researchers) |
| 2011 | **Anticholinergic Loading Scale** [**(18)**](https://www.zotero.org/google-docs/?YhrjjB) **(ALS)** | Included | Australia/Thailand | 293 | numerical, discrete, from 0 to 3 | No | Expert committee (2 psychiatrists, 1 clinical pharmacologist,1 geriatrician) |
| 2012 | **Whalley’s scale** [**(19)**](https://www.zotero.org/google-docs/?KbRXyk) | Excluded because unavailable upon request to the authors. | Scotland |  |  |  | Literature review of serum anticholinergic activity |
| 2013 | **Durán’s Scale** [**(20)**](https://www.zotero.org/google-docs/?QORvGl) **(Duran)** | Included and recoded:  We used the same scoring as that of Lisibach et al. [(21)](https://www.zotero.org/google-docs/?GDhJj5) and coded “0” as 0, “0 or 1” as 0.5, drugs listed in “Table 4” as 1, “weak” as 2, and “strong” as 3. | Ecuador, Belgium | 250 | categorical (“0”, “0 or 1”, “strong discrepancies”, “weak”, “strong”) | No | Review of pre-existing scales by several authors (ADS, ARS, ABC, CrAS, Chew 2008, AAS and ALS) |
| 2014 | **Drug Burden Index revised by the WHO** [**(22)**](https://www.zotero.org/google-docs/?h0pZez) **(DBI-WHO)** | Included (original version)  Note: We used the list of anticholinergic drugs used by Dispennette et al. [(23)](https://www.zotero.org/google-docs/?69EXbQ) because they were the first to provide a list of drugs for the DBI-WHO. | France | NA (formula) | numerical, continuous, between 0 and 1 | Yes | Revised version of the DBI |
| 2014 | **modified Anticholinergic Risk Scale** [**(24)**](https://www.zotero.org/google-docs/?joRztc) **(mARS)** | Included | UK/ Australia | 61 | numerical, discrete, from 1 to 3 | No | Review of a pre-existing scale by a distinct research team (ARS) |
| 2015 | **Delirogenic Risk Scale** [**(25)**](https://www.zotero.org/google-docs/?FBeNtk) **(DRS)** | Included | Germany | 106 | numerical, discrete, from 1 to 4 | No | Review of a pre-existing scale by a distinct research team (in cases of discrepancies, the scale of Chew was preferred, followed by ADS and ABC) |
| 2015 | **Salahudeen’s scale** [**(26)**](https://www.zotero.org/google-docs/?zCMEdm) **(Salahudeen)** | Included and recoded:  We took the minimum score when several scores were proposed, for example, “1 or 2” was coded as 1 and “2 or 3” was coded as 2. | New Zealand | 195 | numerical (discrete) and partially categorical (“1 or 2”, “1 or 3”, “2 or 3”, “1, 2 or 3”) | No | Review of pre-existing scales by a distinct research team (ADS, ABC, CrAS, ARS, ACB, AAS, ACL) |
| 2016 | **Drug Delirium Scale** [**(27)**](https://www.zotero.org/google-docs/?S9NyUC) **(DDS)** | Excluded because it does not exclusively evaluate anticholinergic properties. | Canada | 96 | numerical, discrete, from 1 to 2 | No | Expert committee consensus (2 geriatric physicians and 2 geriatric pharmacists) |
| 2016 | **Anticholinergic Effect on Cognition** [**(28)**](https://www.zotero.org/google-docs/?s3YcBq) **(AEC)** | Included (the scale is updated regularly on the website https://medichec.com/; we used the online version available in November 2022) | UK | 165 in the original version, 237 in the update | numerical, discrete, from 0 to 3 | No | Literature review of in vitro anticholinergic activity followed by application of a scoring rule |
| 2017 | **Anticholinergic Impregnation Scale** [**(29)**](https://www.zotero.org/google-docs/?WnEidV) **(AIS)** | Included (its updated version by the same authors [(30)](https://www.zotero.org/google-docs/?Kr1iRL) in 2022)  The AIS scale was provided with the drugs' ability to penetrate the brain-blood barrier (BBB). However, we decided not to include it in the scoring as it pertained to only 4 patients in our sample. | France | 128 in the original version, 187 in the update | numerical, discrete, from 1 to 3 | No | Review of pre-existing scales by a distinct research team (ADS, ABC, CrAS, ARS, ACB, AAS, ACL, AEC, MARANTE, German ACB, Brazilian ADS, Korean ABS) |
| 2017 | **Muscarinic Acetylcholinergic Receptor ANTagonist Exposure Scale** [**(31)**](https://www.zotero.org/google-docs/?f8BQo5) **(MARANTE)** | Included (update of the scale received from the authors in October 2022) | The Netherlands, Belgium, Ecuador | 41 in the original version, 102 in the update | numerical, discrete, from 0 to 2 | Yes | Review of a pre-existing scale by a distinct research team (Duran) and implementation of a dose-dependent factor (based on literature review) |
| 2017 | **Anticholinergic Toxicity Scale** [**(32)**](https://www.zotero.org/google-docs/?UdAbRi) **(ATS)** | Included | USA | 25 | numerical, continuous, from 0.6 to 5 | No | Computational modeling (computational receptor binding affinity based on the molecular structures of the medications) |
| 2018 | **German Anticholinergic Burden Scale** [**(33)**](https://www.zotero.org/google-docs/?v83oos) **(German ACB)** | Included | Germany | 504 | numerical, discrete, from 0 to 3 | No | Review of pre-existing scales by a distinct research team (ACB, ADS, ABC, ARS, CrAS, AAS, ALS, Duran scale) |
| 2019 | **Korean Anticholinergic Burden Scale** [**(34)**](https://www.zotero.org/google-docs/?6wrE5H) **(KABS)** | Included | Korea | 494 | numerical, discrete, from 0 to 3 | No | Review of pre-existing scales by a distinct research team (ABS, ARS, ACB, ADS, ALS, CrAS, Chew scale, AAS, ABC) |
| 2019 | **Brazilian Anticholinergic Activity Drug Scale** [**(35)**](https://www.zotero.org/google-docs/?TSXtS3) **(Brazilian ADS)** | Included | Brazil | 125 | numerical, discrete, from 1 to 3 | No | Review of pre-existing scales by a distinct research team (ADS, ABC, CrAS, ARS, Chew scale, ACB, AAS, ALS, AEC, MARANTE, AIS) |
| 2019 | **First modified version of the Anticholinergic Cognitive Burden Scale** [**(36)**](https://www.zotero.org/google-docs/?Qq1uXu) **(mACB1)** | Included | Australia | 82 | numerical, discrete, from 1 to 3 | No | Review of a pre-existing scale by a distinct research team (ACB) |
| 2021 | **Second modified version of the Anticholinergic Cognitive Burden Scale** [**(37)**](https://www.zotero.org/google-docs/?BEhoGD) **(mACB2)** | Included | USA | 59 | numerical, discrete, from 0 to 3 | No | Review of a pre-existing scale by a distinct research team (ACB) |
| 2021 | **CRIDECO Anticholinergic Load Scale** [**(38)**](https://www.zotero.org/google-docs/?AbWKko) **(CALS)** | Included | Spain | 217 | numerical, discrete, from 1 to 3 | No | Review of pre-existing scales by a distinct research team (ADS, ACB, Duran's scale, ARS, Salahuden's scale, German ABS, Korean ABS) |

### **Supplementary material 2 (SM2): covariate selection**

We ran pairwise correlation analyses for the 50 imputed datasets between the score in the Anticholinergic Cognitive Burden Scale (ACB) [(15)](https://www.zotero.org/google-docs/?3STILa), the scale validated by the most studies [(21)](https://www.zotero.org/google-docs/?0G4TEK), and each variable among age, sex, education level, PANSS positive and negative scores, Calgary score, the subtype of schizophrenia spectrum disorder, the number of previous psychotic episodes, the number of hospitalizations, and the Clinical Global Impression scale.

Variables associated with the ACB with a *p*-value < 0.2, i.e., age, PANSS positive score, PANSS negative score, Calgary score, the number of previous psychotic episodes and hospitalizations, and the Clinical Global Impression scale, were used as covariates in the analysis (**Supp. Table 1**).

### **Supplementary material (SM3): selection of the threshold for high anticholinergic burden**

We considered patients to have a high anticholinergic burden if their scores were higher than a cut-off. We used the cut-offs proposed by Lisibach et al. [(39)](https://www.zotero.org/google-docs/?krzlVM), which are described below:

- “The score is ≥ 3” in the ADS, the KABS, the GACB, the mACB2, Chew’s scale, the AIS, the AEC, the CALS, Salahudeen’s scale, Duran’s scale, the BADS, the DRS, the ALS, the ACB, the mACB1, the AAS, the CrAS, the Marante, the mARS, the ARS, the ABC, the ATS, Summers’s scale, and Peters’s scale.
- “The score is ≥ 1” in the DBI-WHO scale (based on the criteria of the DBI scale on the Web Portal Software Anticholinergic Burden Calculator (https://www.anticholinergicscales.es/).
- “The score is ≥ 0.1 mg benztropine equivalents” in the PI scale.
- “The score is ≥ 0.02 mg benztropine equivalents” in the CI scale.

## References

[1. Summers WK. A clinical method of estimating risk of drug induced delirium. Life Sci. 1 mai 1978;22(17):1511‑6.](https://www.zotero.org/google-docs/?DYokNA)

[2. Peters NL. Snipping the thread of life. Antimuscarinic side effects of medications in the elderly. Archives of internal medicine. 1989;2414‑20.](https://www.zotero.org/google-docs/?DYokNA)

[3. Tune L, Carr S, Hoag E, Cooper T. Anticholinergic effects of drugs commonly prescribed for the elderly: potential means for assessing risk of delirium. Am J Psychiatry. oct 1992;149(10):1393‑4.](https://www.zotero.org/google-docs/?DYokNA)

[4. Han L, McCusker J, Cole M, Abrahamowicz M, Primeau F, Elie M. Use of medications with anticholinergic effect predicts clinical severity of delirium symptoms in older medical inpatients. Arch Intern Med. 23 avr 2001;161(8):1099‑105.](https://www.zotero.org/google-docs/?DYokNA)

[5. Carnahan RM, Lund BC, Perry PJ, Culp KR, Pollock BG. The relationship of an anticholinergic rating scale with serum anticholinergic activity in elderly nursing home residents. Psychopharmacol Bull. 2002;36(4):14‑9.](https://www.zotero.org/google-docs/?DYokNA)

[6. Aizenberg D, Sigler M, Weizman A, Barak Y. Anticholinergic burden and the risk of falls among elderly psychiatric inpatients: a 4-year case-control study. Int Psychogeriatr. sept 2002;14(3):307‑10.](https://www.zotero.org/google-docs/?DYokNA)

[7. Minzenberg MJ, Poole JH, Benton C, Vinogradov S. Association of anticholinergic load with impairment of complex attention and memory in schizophrenia. Am J Psychiatry. janv 2004;161(1):116‑24.](https://www.zotero.org/google-docs/?DYokNA)

[8. Carnahan RM, Lund BC, Perry PJ, Pollock BG, Culp KR. The Anticholinergic Drug Scale as a measure of drug-related anticholinergic burden: associations with serum anticholinergic activity. J Clin Pharmacol. déc 2006;46(12):1481‑6.](https://www.zotero.org/google-docs/?DYokNA)

[9. Ancelin ML, Artero S, Portet F, Dupuy AM, Touchon J, Ritchie K. Non-degenerative mild cognitive impairment in elderly people and use of anticholinergic drugs: longitudinal cohort study. BMJ. 25 févr 2006;332(7539):455‑9.](https://www.zotero.org/google-docs/?DYokNA)

[10. Cancelli I, Valentinis L, Merlino G, Valente M, Gigli GL. Drugs with anticholinergic properties as a risk factor for psychosis in patients affected by Alzheimer’s disease. Clin Pharmacol Ther. juill 2008;84(1):63‑8.](https://www.zotero.org/google-docs/?DYokNA)

[11. Hilmer SN, Mager DE, Simonsick EM, Cao Y, Ling SM, Windham BG, et al. A drug burden index to define the functional burden of medications in older people. Arch Intern Med. 23 avr 2007;167(8):781‑7.](https://www.zotero.org/google-docs/?DYokNA)

[12. Han L, Agostini JV, Allore HG. Cumulative anticholinergic exposure is associated with poor memory and executive function in older men. J Am Geriatr Soc. déc 2008;56(12):2203‑10.](https://www.zotero.org/google-docs/?DYokNA)

[13. Cao YJ, Mager DE, Simonsick EM, Hilmer SN, Ling SM, Windham BG, et al. Physical and cognitive performance and burden of anticholinergics, sedatives, and ACE inhibitors in older women. Clin Pharmacol Ther. mars 2008;83(3):422‑9.](https://www.zotero.org/google-docs/?DYokNA)

[14. Rudolph JL, Salow MJ, Angelini MC, McGlinchey RE. The anticholinergic risk scale and anticholinergic adverse effects in older persons. Arch Intern Med. 10 mars 2008;168(5):508‑13.](https://www.zotero.org/google-docs/?DYokNA)

[15. Boustani M, Campbell N, Munger S, Fox C. Impact of anticholinergics on the aging brain: A review and practical application. Aging Health. 2008;311‑20.](https://www.zotero.org/google-docs/?DYokNA)

[16. Chew ML, Mulsant BH, Pollock BG, Lehman ME, Greenspan A, Mahmoud RA, et al. Anticholinergic activity of 107 medications commonly used by older adults. J Am Geriatr Soc. juill 2008;56(7):1333‑41.](https://www.zotero.org/google-docs/?DYokNA)

[17. Ehrt U, Broich K, Larsen JP, Ballard C, Aarsland D. Use of drugs with anticholinergic effect and impact on cognition in Parkinson’s disease: a cohort study. J Neurol Neurosurg Psychiatry. févr 2010;81(2):160‑5.](https://www.zotero.org/google-docs/?DYokNA)

[18. Sittironnarit G, Ames D, Bush AI, Faux N, Flicker L, Foster J, et al. Effects of anticholinergic drugs on cognitive function in older Australians: results from the AIBL study. Dement Geriatr Cogn Disord. 2011;31(3):173‑8.](https://www.zotero.org/google-docs/?DYokNA)

[19. Whalley LJ, Sharma S, Fox HC, Murray AD, Staff RT, Duthie AC, et al. Anticholinergic drugs in late life: adverse effects on cognition but not on progress to dementia. J Alzheimers Dis JAD. 2012;30(2):253‑61.](https://www.zotero.org/google-docs/?DYokNA)

[20. Durán CE, Azermai M, Vander Stichele RH. Systematic review of anticholinergic risk scales in older adults. Eur J Clin Pharmacol. juill 2013;69(7):1485‑96.](https://www.zotero.org/google-docs/?DYokNA)

[21. Lisibach A, Benelli V, Ceppi MG, Waldner-Knogler K, Csajka C, Lutters M. Quality of anticholinergic burden scales and their impact on clinical outcomes: a systematic review. Eur J Clin Pharmacol. févr 2021;77(2):147‑62.](https://www.zotero.org/google-docs/?DYokNA)

[22. Dauphinot V, Faure R, Omrani S, Goutelle S, Bourguignon L, Krolak-Salmon P, et al. Exposure to anticholinergic and sedative drugs, risk of falls, and mortality: an elderly inpatient, multicenter cohort. J Clin Psychopharmacol. oct 2014;34(5):565‑70.](https://www.zotero.org/google-docs/?DYokNA)

[23. Dispennette R, Elliott D, Nguyen L, Richmond R. Drug Burden Index score and anticholinergic risk scale as predictors of readmission to the hospital. Consult Pharm J Am Soc Consult Pharm. mars 2014;29(3):158‑68.](https://www.zotero.org/google-docs/?DYokNA)

[24. Sumukadas D, McMurdo MET, Mangoni AA, Guthrie B. Temporal trends in anticholinergic medication prescription in older people: repeated cross-sectional analysis of population prescribing data. Age Ageing. juill 2014;43(4):515‑21.](https://www.zotero.org/google-docs/?DYokNA)

[25. Hefner G, Shams MEE, Wenzel‐Seifert K, Fellgiebel A, Falter T, Haen E, et al. Rating The Delirogenic Potential of Drugs for Prediction of Side Effects in Elderly Psychiatric Inpatients. In 2015.](https://www.zotero.org/google-docs/?DYokNA)

[26. Salahudeen MS, Duffull SB, Nishtala PS. Anticholinergic burden quantified by anticholinergic risk scales and adverse outcomes in older people: a systematic review. BMC Geriatr. 25 mars 2015;15:31.](https://www.zotero.org/google-docs/?DYokNA)

[27. Nguyen PPL, Payot I, Latour J. Drug Delirium Scale (DDS): A tool to evaluate drugs as a risk factor for Delirium. Int J Innov Res Med Sci. 2016;232‑7.](https://www.zotero.org/google-docs/?DYokNA)

[28. Bishara D, Harwood D, Sauer J, Taylor DM. Anticholinergic effect on cognition (AEC) of drugs commonly used in older people. Int J Geriatr Psychiatry. juin 2017;32(6):650‑6.](https://www.zotero.org/google-docs/?DYokNA)

[29. Briet J, Javelot H, Heitzmann E, Weiner L, Lameira C, D’Athis P, et al. The anticholinergic impregnation scale: Towards the elaboration of a scale adapted to prescriptions in French psychiatric settings. Therapie. sept 2017;72(4):427‑37.](https://www.zotero.org/google-docs/?DYokNA)

[30. Javelot H, Meyer G, Becker G, Post G, Runge V, Pospieszynski P, et al. [Anticholinergic scales: Use in psychiatry and update of the anticholinergic impregnation scale]. L’Encephale. juin 2022;48(3):313‑24.](https://www.zotero.org/google-docs/?DYokNA)

[31. Klamer TT, Wauters M, Azermai M, Durán C, Christiaens T, Elseviers M, et al. A Novel Scale Linking Potency and Dosage to Estimate Anticholinergic Exposure in Older Adults: the Muscarinic Acetylcholinergic Receptor ANTagonist Exposure  Scale. Basic Clin Pharmacol Toxicol. juin 2017;120(6):582‑90.](https://www.zotero.org/google-docs/?DYokNA)

[32. Xu D, Anderson HD, Tao A, Hannah KL, Linnebur SA, Valuck RJ, et al. Assessing and predicting drug-induced anticholinergic risks: an integrated computational approach. Ther Adv Drug Saf. nov 2017;8(11):361‑70.](https://www.zotero.org/google-docs/?DYokNA)

[33. Kiesel EK, Hopf YM, Drey M. An anticholinergic burden score for German prescribers: score development. BMC Geriatr. 11 oct 2018;18(1):239.](https://www.zotero.org/google-docs/?DYokNA)

[34. Jun K, Hwang S, Ah YM, Suh Y, Lee JY. Development of an Anticholinergic Burden Scale specific for Korean older adults. Geriatr Gerontol Int. juill 2019;19(7):628‑34.](https://www.zotero.org/google-docs/?DYokNA)

[35. Nery RT, Reis AMM. Development of a Brazilian anticholinergic activity drug scale. Einstein Sao Paulo Braz. 1 avr 2019;17(2):eAO4435.](https://www.zotero.org/google-docs/?DYokNA)

[36. Kable A, Fullerton A, Fraser S, Palazzi K, Hullick C, Oldmeadow C, et al. Comparison of Potentially Inappropriate Medications for People with Dementia at Admission and Discharge during An Unplanned Admission to Hospital: Results from  the SMS Dementia Study. Healthc Basel Switz. 9 janv 2019;7(1).](https://www.zotero.org/google-docs/?DYokNA)

[37. Joshi YB, Thomas ML, Braff DL, Green MF, Gur RC, Gur RE, et al. Anticholinergic Medication Burden-Associated Cognitive Impairment in Schizophrenia. Am J Psychiatry. 1 sept 2021;178(9):838‑47.](https://www.zotero.org/google-docs/?DYokNA)

[38. Ramos H, Moreno L, Pérez-Tur J, Cháfer-Pericás C, García-Lluch G, Pardo J. CRIDECO Anticholinergic Load Scale: An Updated Anticholinergic Burden Scale. Comparison with the ACB Scale in Spanish Individuals with Subjective Memory  Complaints. J Pers Med. 3 févr 2022;12(2).](https://www.zotero.org/google-docs/?DYokNA)

[39. Lisibach A, Gallucci G, Beeler PE, Csajka C, Lutters M. High anticholinergic burden at admission associated with in-hospital mortality in older patients: A comparison of 19 different anticholinergic burden scales. Basic Clin Pharmacol Toxicol. févr 2022;130(2):288‑300.](https://www.zotero.org/google-docs/?DYokNA)
