# Supplementary material for: Comparative analysis of anticholinergic burden scales to explain iatrogenic cognitive impairment in schizophrenia: results from the multicenter FACE-SZ cohort
Source: Front Pharmacol. 2024 Jun 12;15:1403093. doi: 10.3389/fphar.2024.1403093 (PMC11200119; doi:10.3389/fphar.2024.1403093)
Supplement: Supplementary file 5 [file Image1.PDF]

Supplementary Figure 1. Impression of the website page of the *U.S. Food & Drug Administration* provinding the list of atypical antipsychotics (https://www.fda.gov/drugs/postmarket-drug-safety-information-patients-and-providers/atypical-antipsychotic-drugs-information).

Atypical Antipsychotic Drugs Information

Aripiprazole (marketed as Abilify)

Asenapine Maleate (marketed as Saphris)

Clozapine (marketed as Clozaril)

Iloperidone (marketed as Fanapt)

Lurasidone (marketed as Latuda)

Olanzapine (marketed as Zyprexa)

Olanzapine/Fluoxetine (marketed as Symbyax)

Paliperidone (marketed as Invega)

Quetiapine (marketed as Seroquel)

Risperidone (marketed as Risperdal)

Ziprasidone (marketed as Geodon)

To report any unexpected adverse or serious events associated with the use of these drugs, please contact the FDA MedWatch program using the information at the bottom of this page.

Related Information

- FDA Drug Safety Communication: FDA warns about rare but serious skin reactions with mental health drug olanzapine (Zyprexa, Zyprexa Zydis, Zyprexa Relprevv, and Symbyax) (/drugs/drug-safety-and-availability/fda-drug-safety-communication-fda-warns-about-rare-serious-skin-reactions-mental-health-drug)
- FDA Drug Safety Communication: FDA warns about new impulse-control problems associated with mental health drug aripiprazole (Abilify, Abilify Maintena, Aristada) (/drugs/drug-safety-and-availability/fda-drug-safety-communication-fda-warns-about-new-impulse-control-problems-associated-mental-health)
- FDA Drug Safety Communication: FDA modifies monitoring for neutropenia associated with schizophrenia drug clozapine; approves new shared REMS program for all clozapine medicines (/drugs/drug-safety-and-availability/fda-drug-safety-communication-fda-modifies-monitoring-neutropenia-associated-schizophrenia-medicine)
- FDA Drug Safety Communication: FDA review of study sheds light on two deaths associated with the injectable schizophrenia drug Zyprexa Relprevv (olanzapine pamoate) (/drugs/drug-safety-and-availability/fda-drug-safety-communication-fda-review-study-sheds-light-two-deaths-associated-injectable)
- FDA Drug Safety Communication: FDA reporting mental health drug ziprasidone (Geodon) associated with rare but potentially fatal skin reactions (/drugs/drug-safety-and-availability/fda-drug-safety-communication-fda-reporting-mental-health-drug-ziprasidone-geodon-associated-rare)
- FDA Drug Safety Communication: FDA is investigating two deaths following injection of long-acting antipsychotic Zyprexa Relprevv (olanzapine pamoate) (https://wayback.archive-it.org/7993/20170722185807/https:/www.fda.gov/Drugs/DrugSafety/ucm356971.htm) (http://www.fda.gov/about-fda/website-policies/website-disclaimer)
- FDA Drug Safety Communication: Serious allergic reactions reported with the use of Saphris (asenapine maleate) (/drugs/drug-safety-and-availability/fda-drug-safety-communication-serious-allergic-reactions-reported-use-saphris-asenapine-maleate)
- FDA Drug Safety Communication: Medication errors resulting from confusion between risperidone (Risperdal) and ropinirole (Requip) (/drugs/drug-safety-and-availability/fda-drug-safety-communication-medication-errors-resulting-confusion-between-risperidone-risperdal)
- FDA Drug Safety Communication: Antipsychotic drug labels updated on use during pregnancy and risk of abnormal muscle movements and withdrawal symptoms in newborns (/drugs/drug-safety-and-availability/fda-drug-safety-communication-antipsychotic-drug-labels-updated-use-during-pregnancy-and-risk)
- Information for Healthcare Professionals: Olanzapine/fluoxetine (marketed as Symbyax) (https://wayback.archive-it.org/7993/20170406044248/https:/www.fda.gov/Drugs/DrugSafety/PostmarketDrugSafetyInformationforPatientsandProviders/DrugSafetyInformationfor [illegible] (http://www.fda.gov/about-fda/website-policies/website-disclaimer)
- Historical Information on Atypical Antipsychotic Drugs (https://wayback.archive-it.org/7993/20170406044252/https:/www.fda.gov/Drugs/DrugSafety/PostmarketDrugSafetyInformationforPatientsandProviders/ucm149296.htm) (http://www.fda.gov/about-fda/website-policies/website-disclaimer)
- Information on Conventional Antipsychotics (https://wayback.archive-it.org/7993/20170722033234/https:/www.fda.gov/Drugs/DrugSafety/PostmarketDrugSafetyInformationforPatientsandProviders/ucm107211.htm) (http://www.fda.gov/about-fda/website-policies/website-disclaimer)

Labeling and Regulatory History from Drugs@FDA

- Aripiprazole (marketed as Abilify) Approval History and Labeling Information (http://www.accessdata.fda.gov/scripts/cder/drugsatfda/index.cfm?fuseaction=Search.SearchAction&SearchTerm=abilify&SearchType=BasicSearch)
- Asenapine Maleate (marketed as Saphris) Prescribing and Label Information (http://www.accessdata.fda.gov/scripts/cder/drugsatfda/index.cfm?fuseaction=Search.SearchAction&searchTerm=022117&SearchType=BasicSearch)
- Clozapine (marketed as Clozaril) Approval History and Labeling Information (http://www.accessdata.fda.gov/scripts/cder/drugsatfda/index.cfm?fuseaction=Search.SearchAction&SearchTerm=clozapine&SearchType=BasicSearch)
- Iloperidone (marketed as Fanapt) Approval History and Labeling Information (http://www.accessdata.fda.gov/scripts/cder/drugsatfda/index.cfm?fuseaction=Search.SearchAction&SearchTerm=Fanapt&SearchType=BasicSearch)
- Lurasidone (marketed as Latuda) Approval History and Labeling information (http://www.accessdata.fda.gov/scripts/cder/drugsatfda/index.cfm?fuseaction=Search.SearchAction&SearchTerm=Latuda&SearchType=BasicSearch)
- Olanzapine (marketed as Zyprexa) Approval History and Labeling Information (http://www.accessdata.fda.gov/scripts/cder/drugsatfda/index.cfm?fuseaction=Search.SearchAction&SearchTerm=zyprexa&SearchType=BasicSearch)
- Olanzapine/Fluoxetine (marketed as Symbyax) Prescribing and Label Information (http://www.accessdata.fda.gov/scripts/cder/drugsatfda/index.cfm?fuseaction=Search.SearchAction&SearchTerm=symbyax&SearchType=BasicSearch)
- Paliperidone (marketed as Invega) Approval History and Labeling Information (http://www.accessdata.fda.gov/scripts/cder/drugsatfda/index.cfm?fuseaction=Search.SearchAction&SearchTerm=Invega&SearchType=BasicSearch)
- Quetiapine (marketed as Seroquel) Prescribing and Label Informaiton (http://www.accessdata.fda.gov/scripts/cder/drugsatfda/index.cfm?fuseaction=Search.SearchAction&SearchTerm=quetiapine&SearchType=BasicSearch)
- Risperidone (marketed as Risperdal) Prescribing and Label Information (http://www.accessdata.fda.gov/scripts/cder/drugsatfda/index.cfm?fuseaction=Search.SearchAction&SearchTerm=risperidone&SearchType=BasicSearch)
- Ziprasidone (marketed as Geodon) Prescribing and Label Information (http://www.accessdata.fda.gov/scripts/cder/drugsatfda/index.cfm?fuseaction=Search.SearchAction&SearchTerm=ziprasidone&SearchType=BasicSearch)
